# 2b or not 2b? 2bRAD is an effective alternative to ddRAD for phylogenomics

**DOI:** 10.1002/ece3.9842

**Published:** 2023-03-08

**Authors:** E. Anne Chambers, Rebecca D. Tarvin, Juan C. Santos, Santiago R. Ron, Mileidy Betancourth‐Cundar, David M. Hillis, Mikhail V. Matz, David C. Cannatella

**Affiliations:** ^1^ Department of Integrative Biology and Biodiversity Center University of Texas at Austin Austin Texas USA; ^2^ Department of Environmental Science, Policy, and Management and Museum of Vertebrate Zoology University of California Berkeley Berkeley California USA; ^3^ Department of Integrative Biology and Museum of Vertebrate Zoology University of California Berkeley Berkeley California USA; ^4^ Department of Biological Sciences St John's University New York New York USA; ^5^ Museo de Zoología, Escuela de Ciencias Biológicas Pontificia Universidad Católica del Ecuador Quito Ecuador; ^6^ Departamento de Ciencias Biológicas Universidad de los Andes Bogotá Colombia

**Keywords:** Dendrobatidae, missing data, phylogenetic signal, Ranidae, restriction‐site‐associated DNA sequencing

## Abstract

Restriction‐site‐associated DNA sequencing (RADseq) has become an accessible way to obtain genome‐wide data in the form of single‐nucleotide polymorphisms (SNPs) for phylogenetic inference. Nonetheless, how differences in RADseq methods influence phylogenetic estimation is poorly understood because most comparisons have largely relied on conceptual predictions rather than empirical tests. We examine how differences in ddRAD and 2bRAD data influence phylogenetic estimation in two non‐model frog groups. We compare the impact of method choice on phylogenetic information, missing data, and allelic dropout, considering different sequencing depths. Given that researchers must balance input (funding, time) with output (amount and quality of data), we also provide comparisons of laboratory effort, computational time, monetary costs, and the repeatability of library preparation and sequencing. Both 2bRAD and ddRAD methods estimated well‐supported trees, even at low sequencing depths, and had comparable amounts of missing data, patterns of allelic dropout, and phylogenetic signal. Compared to ddRAD, 2bRAD produced more repeatable datasets, had simpler laboratory protocols, and had an overall faster bioinformatics assembly. However, many fewer parsimony‐informative sites per SNP were obtained from 2bRAD data when using native pipelines, highlighting a need for further investigation into the effects of each pipeline on resulting datasets. Our study underscores the importance of comparing RADseq methods, such as expected results and theoretical performance using empirical datasets, before undertaking costly experiments.

## Graphical abstract

Our manuscript examines how differences in data from two RADseq methods – ddRAD and 2bRAD – influence phylogenetic estimation in two non‐model frog groups. We compare the impact of method choice on phylogenetic information, missing data, and allelic dropout, considering different sequencing depths. Both 2bRAD and ddRAD methods estimated well‐supported trees, even at low sequencing depths, and had comparable amounts of missing data, patterns of allelic dropout, and phylogenetic signal. Given that researchers must balance input (funding, time) with output (amount and quality of data), we also provide comparisons of laboratory effort, computational time, monetary costs, and the repeatability of library preparation and sequencing, finding that 2bRAD produced more repeatable datasets, had simpler laboratory protocols, and had an overall faster bioinformatics assembly.

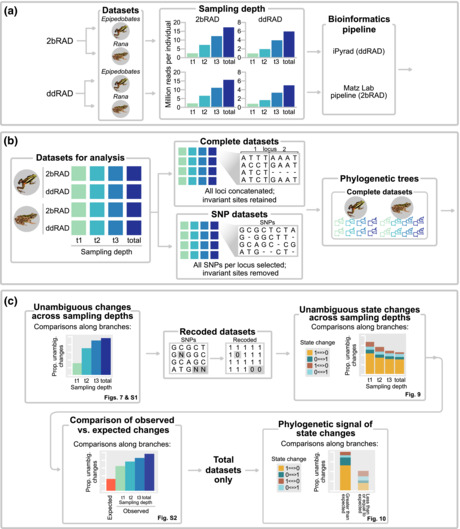

## 1 INTRODUCTION

Although first introduced for genotyping and population genomics studies, genome‐wide reduced representation datasets have become increasingly common for phylogeny estimation at deeper timescales (Cariou et al., 2013; DaCosta & Sorenson, 2016; Eaton et al., 2017; Leaché & Oaks, 2017; Rubin et al., 2012). These datasets are commonly generated using restriction‐site‐associated DNA sequencing methods (RADseq; Davey & Blaxter, 2010), which rely on restriction endonucleases to fragment the genome, followed by sequencing a small portion (usually 0.1%–1%) of the genome to reduce sequencing costs. PCR amplification and sequencing of these fragments generate thousands of loci with single‐nucleotide polymorphisms (SNPs) across the entire genomes of focal taxa and are useful for population genetics analyses and phylogeny estimation.

With the emerging popularity of RADseq for phylogenetics, there has been a corresponding desire to understand how characteristics of SNP data, such as missing data and phylogenetic signal, affect phylogenetic performance (Eaton et al., 2017; Huang & Knowles, 2016; Leaché, Banbury, et al., 2015). However, few studies have explored how differences among RADseq methods, such as fragment size, enzyme type, and number of SNPs recovered, influence dataset assembly and phylogenetic estimates. To date, most comparisons between RADseq methods have relied largely on computational or modeling approaches using simulated data (Andrews et al., 2016; Catchen et al., 2017; Eaton et al., 2017; Flanagan & Jones, 2018; Lowry et al., 2017). Here, we perform a direct empirical examination of how data produced by two common methods – ddRAD (double‐digest RADseq; Peterson et al., 2012) and 2bRAD (Wang et al., 2012) – influence phylogenetic estimation.

The ddRAD method uses two restriction enzymes with different cutting frequencies to cleave the genome into fragments. Next, fragments of a desired size range are retained (size selection) to ensure efficiency in sequencing. By altering the enzyme pair and selected fragment size, the desired percentage of the genome can be targeted for sequencing. Because of these advantages, ddRAD rapidly became the standard RADseq method for population genetics (Halbritter et al., 2019; Mynhardt et al., 2020; Puritz et al., 2014) and phylogenetic estimation (Devitt et al., 2019; Leaché, Chavez, et al., 2015) for species lacking a reference genome.

In contrast, the 2bRAD method employs a single type‐IIB restriction enzyme that cleaves DNA on either side of its recognition site. No size‐selection step is necessary as fragments are all the same length (36 bp in the case of *Bcg*I) and sequencing is expected to recover all fragments (although it is possible to restrict the sequencing to a subset of all fragments through modification of ligation adaptors; see Barbanti et al., 2020; Wang et al., 2012). Apart from the original publication in which the method was used on humans (Wang et al., 2012), few vertebrate groups have been studied with 2bRAD, including fishes, mice, and turtles (e.g., Barbanti et al., 2020; Borrego et al., 2022; Cui et al., 2018; Manuzzi et al., 2019), and its use in phylogenetics is limited (but see Seetharam & Stuart, 2013).

In theory, we would expect ddRAD to outperform 2bRAD in phylogenetic reconstruction, because the shorter and invariant length of 2bRAD fragments might result in incorrectly clustering paralogs into the same putative locus, potentially resulting in less phylogenetic signal (Andrews et al., 2016). On the contrary, because typical 2bRAD library preparation has no size‐selection step, it presumably recovers every fragment across the genome with the selected recognition site, which means that with deep sequencing, all loci could theoretically be recovered. This would result in better repeatability across libraries, although it could also be problematic in organisms with large genomes (Andrews et al., 2016). Finally, the shorter locus lengths of 2bRAD may be preferable when working with samples with degraded DNA (Barbanti et al., 2020).

Here, we compare ddRAD and 2bRAD sequencing from the same specimens from two frog clades. We first examine differences in sequence assembly between ddRAD and 2bRAD datasets, including dataset rarefaction to approximate differing sequencing depths. Then we ask, what are the advantages and disadvantages of each method for phylogenetic inference? We answer this question by using both ddRAD and 2bRAD datasets to estimate phylogenies and to measure phylogenetic signal, levels of missing data, and allelic dropout. Finally, we explore practical aspects including differences in cost, effort, and the reproducibility of libraries.

## 2 MATERIALS AND METHODS

### 2.1 Sample selection, sequencing, and assembly

**FIGURE 1:**
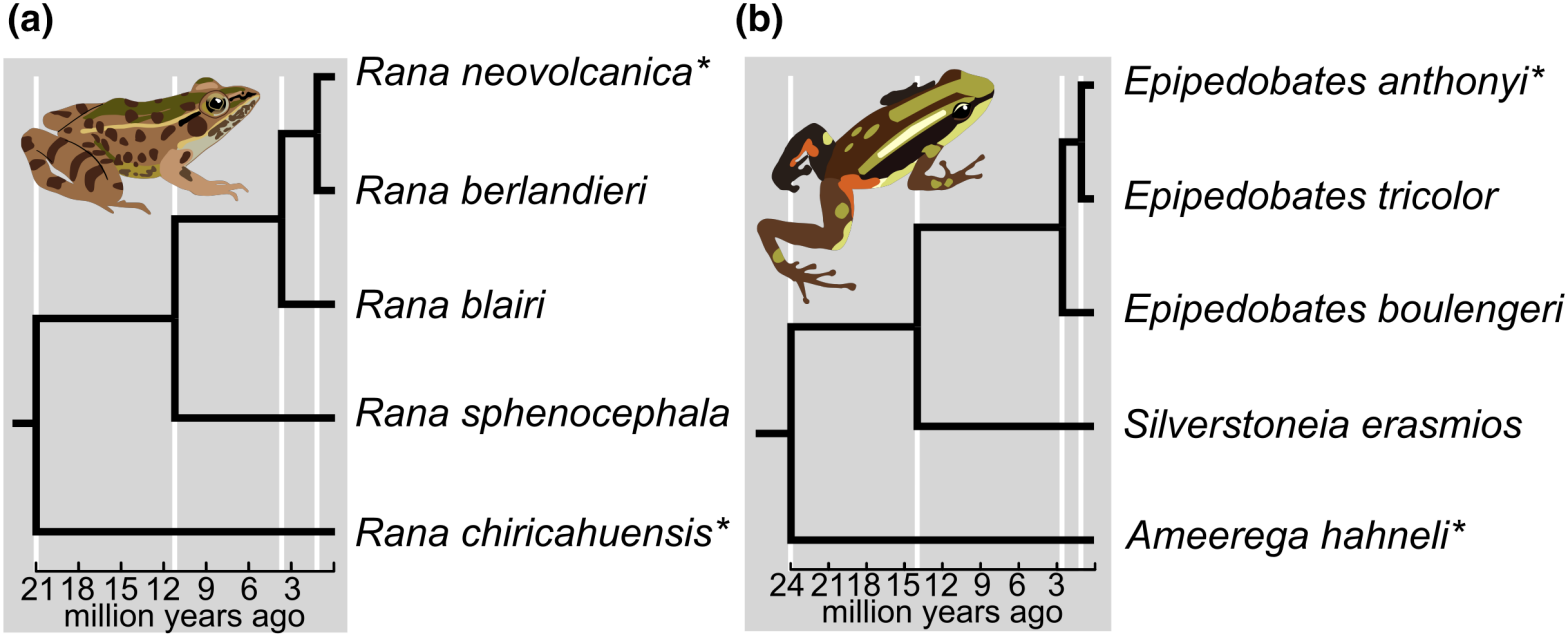
Chronograms of target species from (a) *Rana* (Yuan et al., 2016) and (b) *Epipedobates* (Santos et al., 2009). Two specimens were sequenced from each species. Asterisks indicate that one specimen was sequenced twice as a replicate. Illustrated species: *Epipedobates tricolor*, *Rana berlandieri* (illustrations by EAC).

We selected species from two distantly related frog clades under investigation within our labs: five species of *Rana* (Ranidae) and five species of poison frogs (Dendrobatidae), including three *Epipedobates* species and two close relatives (*Silverstoneia erasmios* and *Ameerega hahneli*). Although the poison frogs include three genera, for brevity we refer to this clade by the name of the ingroup clade, *Epipedobates*. Two individuals of each species were chosen for sequencing, yielding 20 samples. We selected species for each clade such that divergence times were comparable; that is, the ratio of shallowest node age (1.25 million years ago [Ma] in *Rana* and 1.0 Ma in *Epipedobates*) to deepest node age (21 Ma/24 Ma; *Rana*/*Epipedobates*) was similar (Figure 1 and Table S1; Santos et al., 2009; Yuan et al., 2016). DNA was extracted from liver tissue using Qiagen DNeasy blood and tissue kits (Qiagen). Prior to library preparation, DNA was quantified using the dsDNA high‐sensitivity assay on a Qubit 3.0 fluorometer (Life Technologies).

#### 2.1.1 ddRAD sequencing

To determine appropriate restriction enzyme combinations and insert size for ddRAD protocols, 500 ng of DNA for two samples from each clade (*R. chiricahuensis* and *E. anthonyi*) was double digested with four enzyme pairs: *Sph*I + *Eco*RI, *Eco*RI + *Msp*I, *Sph*I + *Mlu*Cl, and *Sph*I + *Msp*I (New England BioLabs), cleaned using handmade Serapure beads (see Rohland & Reich, 2012), and sent to the University of Texas at Austin Genomics Sequencing and Analysis Facility (GSAF) for fragment visualization using an Agilent 2100 Bioanalyzer (Agilent) and standard 2100 Expert Software. We selected the *Sph*I + *Mlu*Cl enzyme combination for both *Epipedobates* and *Rana* because they sheared reasonable subsets of the genomes (~1%) at a size range amenable to Illumina sequencing technology (~300 nt). Based on our Bioanalyzer results, we aimed to recover 0.98% of the genome in *Epipedobates* (size selection window: 275–325 nt; $\bar{x}$ = 291 nt) and 1.21% of the genome in *Rana* (size selection window: 300–350 nt; $\bar{x}$ = 314 nt). We estimated the genome size of *Epipedobates* as 9GB, based on the upper limit for the dendrobatid *Oophaga* (Rogers et al., 2018), and 6GB for *Rana catesbeiana* following Hammond et al. (2017). To target a coverage depth of 20×, we requested 7.27 and 5.55 million paired‐end reads (2 × 150 paired‐end reads) per sample for *Epipedobates* and *Rana*, respectively (Table 1; see also Supporting Information). Preliminary data now suggest that *Epipedobates* genomes are closer to 6GB in size (R. D. Tarvin, *unpublished data*), which would imply that fewer reads could have been requested. Library preparation was performed following Peterson et al. (2012), using handmade Sera‐mag Speedbeads for all but the final bead clean‐up step (in which Dynabeads were used). DNA was quantified using PicoGreen dsDNA quantitation, DNA was standardized, and size selection was accomplished using a Pippin Prep machine (using a 2% cassette). Pooled libraries (total concentrations of 0.92 ng/μL for *Epipedobates* and 1.91 ng/μL for *Rana*) were then sequenced at the GSAF on an Illumina HiSeq 4000.

**TABLE 1:** Results of assembly pipeline for complete dataset (*total* sampling depth).

Dataset	Estimated proportion of genome sequenced^a^ (%)	Requested raw reads (M/ind)	Obtained raw reads (M/ind)	Avg. read depth/ind^b^	Missing data (%)^c^	Total sites	Total loci	Total SNPs	Total PIs^d^	SNPs per locus	SNPs per site	PIs per SNP	PIs per locus
***Epipedobates***													
2bRAD	0.053	25.0	15.6	10.1 (1.89–16.2)	50.6 (15.7–89.8)	3,208,050	89,952	63,070	8196	0.70	0.02	0.13	0.09
ddRAD	0.059	7.3	6.7	21.4 (1.76–40.2)	56.3 (24.2–95.0)	3,558,310	32,371	208,428	73,187	6.44	0.06	0.35	2.26
***Rana***													
2bRAD	0.15	16.7	12.5	9.88 (3.29–18.0)	44.3 (20.3–73.8)	9,133,414	255,197	161,952	19,281	0.63	0.02	0.12	0.08
ddRAD	0.077	5.6	5.8	18.1 (7.64–28.4)	43.6 (30.2–60.3)	8,312,261	75,393	381,817	149,816	5.06	0.05	0.39	1.99

*Note*: Requested reads and obtained reads are the total number of raw reads divided by the number of individuals (10, not including the replicate samples); these values assume equal numbers of reads were obtained across individuals. Minimum and maximum values are indicated in parentheses.

^a^ Post‐processing number of sites (without missing data) for ingroup taxa: *E. anthonyi* 1 and *R. berlandieri* 2, divided by the estimated genome sizes for *Epipedobates* (9GB) and *Rana* (6GB).

^b^ Average of the read depth per individual (across sites), obtained from the vcf of the final assembly.

^c^ Proportion of missing cells in SNP datasets.

^d^ Parsimony‐informative sites; SNPs, single‐nucleotide polymorphisms.

#### 2.1.2 2bRAD sequencing

Using the *Bcg*I enzyme, we digested the same four samples used for the ddRAD test digestion. The Bioanalyzer analysis showed no obvious peak at the size of the 2bRAD insert (36 bp), which is close to the smallest fragment in the ladder of Bioanalyzer gels (35 bp). Therefore, it was difficult to quantify the amount of the genome digested, and we conservatively estimated that the *Bcg*l enzyme cut 0.5% of the genome (based on estimates from previous vertebrates on which this enzyme had been tested). Using the same genome size estimates and depth of coverage (20×) as with ddRAD, we aimed to obtain 25 and 17 million 50‐bp single‐end reads per sample for *Epipedobates* and *Rana*, respectively (Table 1; see Supporting Information for calculations). Extracted DNA was cleaned using Zymo Genomic DNA Clean & Concentrator (Zymo Research), and 100 ng of cleaned DNA was then digested using the *Bcg*I enzyme (New England BioLabs). All libraries were prepared using protocols developed by the Matz lab (see https://github.com/z0on/2bRAD_denovo for the most current protocols and Supporting Information for the protocol used here). Following ligation, libraries were pooled together and then run on an agarose gel; the target 176 bp band was excised manually and purified using agarose gel extractions. Final pooled libraries with total concentrations of 0.22 ng/μL for *Epipedobates* and 0.28 ng/μL for *Rana* were sequenced at the GSAF on an Illumina HiSeq 4000.

#### 2.1.3 Rarefaction of sequencing depths by sampling

**FIGURE 2:**
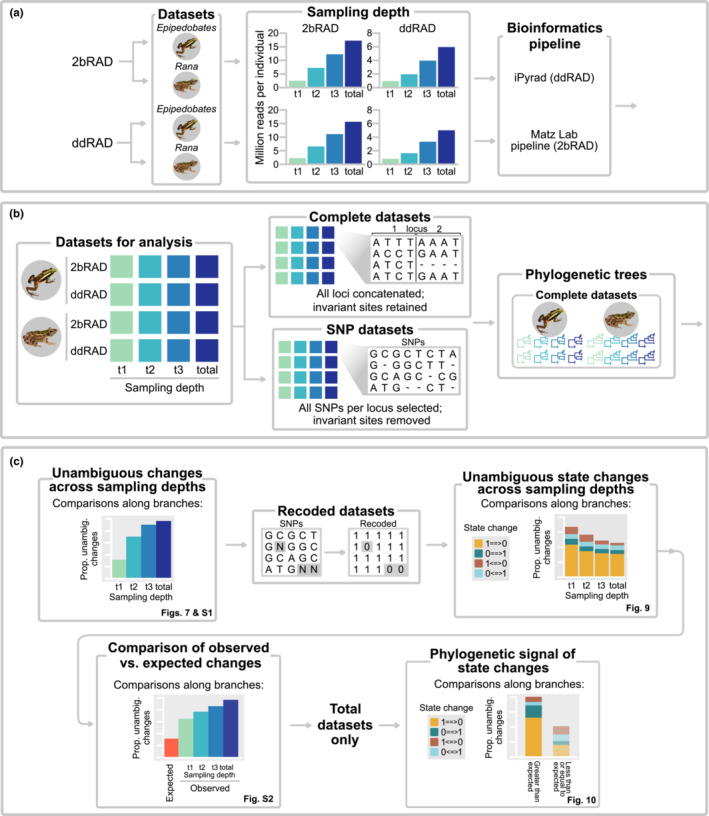
Flowchart of methods. (a) To simulate varying sequencing depths (*t1*, *t2*, and *t3*), we randomly subsampled the complete (*total*) datasets. This produced 16 datasets, 8 for each RADseq method and 1 for each clade. (b) Phylogenetic reconstructions were run using complete datasets (all sites), producing 16 trees. (c) SNP datasets were used to calculate unambiguous changes along branches of trees to quantify phylogenetic information. Allelic dropout was quantified using binary‐recoded (1/0 for presence/absence) SNP datasets under Dollo parsimony.

In ddRAD studies, typical targets of 1–2 M reads/individual are used to reach a 10× sequencing depth, at which shared locus coverage is high among individuals, and genotypes can be called with confidence, enabling researchers to answer questions at both population and phylogenetic levels (Valencia et al., 2018). To determine the effect of different sequencing depths on phylogenetic inference, we targeted a larger number of reads that is typical and then subsampled these. Although rarefaction is not equivalent to sequencing at different depths, we consider it an adequate proxy. We aimed to recover 5.6–7.3 M reads per individual for ddRAD and 17–25 M reads per individual for 2bRAD to yield data at a depth of 20×, two‐fold more than is typical (see Supporting Information for calculations). We randomly sampled reads (without replacement) at arbitrarily selected proportions to represent different sequencing depths from the *Epipedobates* and *Rana* datasets using the *sample* function of *seqtk* (https://github.com/lh3/seqtk) to yield four sampling depths (*t1*, *t2*, *t3*, and *total*; Figure 2a). At the lowest sampling depth (*t1*), we sampled 14%–16% of the total reads to yield approximately 1 M reads per individual, 33%–42% at depth *t2*, and 66%–71% at depth *t3* (Table 2). We sampled the 2bRAD datasets using the same percentages (Table 2). All sampling occurred prior to data processing or filtering.

**TABLE 2:** Numbers of raw reads (summed over all individuals) for each sampling scheme by method and taxon.

Sampling depth	*Epipedobates*			*Rana*		
	Proportion sampled (%)	ddRAD	2bRAD	Proportion sampled (%)	ddRAD	2bRAD
*t1*	14.5	12,008,757	27,199,735	16.1	11,486,579	24,115,588
*t2*	42.0	34,808,757	78,841,547	32.9	23,486,579	49,309,082
*t3*	71.0	58,808,757	133,201,348	66.4	47,486,579	99,696,069
*Total*	100	82,808,757	187,561,150	100	71,486,579	150,083,057

*Note*: The *total* sampling depth is the total number of reads sequenced.

#### 2.1.4 ddRAD assembly

**FIGURE 3:**
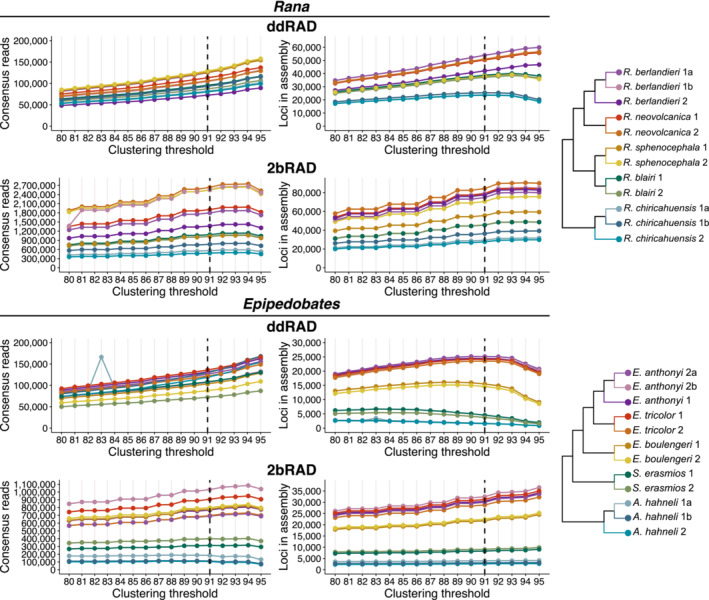
Number of consensus reads (good reads before clustering) and total number of loci (shared between samples) per individual for clustering threshold values from 80% to 95% for *Rana* and *Epipedobates* for ddRAD and 2bRAD datasets. We selected a clustering threshold value of 91% for our analysis (black dotted lines).

Bioinformatics pipelines for 2bRAD and ddRAD were run on the Lonestar 5 system of the Texas Advanced Computing Center (TACC) at the University of Texas at Austin. We used iPyrad v.0.7.23 (Eaton, 2014; Eaton & Overcast, 2020) to assemble the ddRAD datasets of each clade separately. The *total* dataset was used to determine the clustering threshold, which is the percent similarity at which two sequences are considered orthologous and assigned to the same cluster (iPyrad parameter 14, *clust_threshold*). If this parameter is too high (too stringent), loci may be over‐split, meaning that true homologs are interpreted as different loci; however, if the parameter is too low, loci may be under‐split, i.e., paralogs incorrectly clustered into a single locus (Harvey et al., 2015; Ilut et al., 2014). iPyrad applies the clustering threshold parameter during two steps in the pipeline: first, to build clusters within samples, and then, to construct loci among samples. We tested 16 clustering threshold values from 0.80 to 0.95 to assess the effect of this parameter on both steps. As the clustering threshold increases, we expect to see the number of loci assembled per individual increases with the reduced stringency of this parameter. However, when the clustering threshold becomes too high, some putative loci will begin to be erroneously split into different loci, after which the *min_samples_locus* parameter will remove them, resulting in a decrease in the number of useful loci. Thus, the optimal parameter value maximizes the number of loci in individual assembly, thereby also ensuring orthologs are not oversplit. Based on the number of loci obtained for tested clustering values (Figure 3), we chose a conservative clustering threshold of 0.91 for the ddRAD assembly. Although consideration of *Rana* and *Epipedobates* separately would have led us to choose slightly different values for each, we chose a single value because applying similar values of clustering threshold is important for cross‐taxon comparisons (Harvey et al., 2015).

To ensure consistency with the 2bRAD data assembly, consensus reads containing an N (uncalled base) were removed by setting the iPyrad parameter *max_Ns_consens* equal to 0, 0. The number of samples required to share a locus so that it is retained in the assembly (*min_samples_locus*) was set to 4 to maximize the number of loci across all samples. Reads were trimmed to 120 bases, removing the first 5 and last 25 bases, which had lower quality (*trim_reads* = 5, −25, 0, 0). The minimum read length was set to 35 nt (*filter_min_trim_len*). To ensure consistency between final assemblies from each data type, we retained only forward reads and set remaining iPyrad parameters to the default (Table 3).

**TABLE 3:** Assembly pipeline parameter settings used in iPyrad v.0.7.23 (Eaton, 2014) for ddRAD assembly and the Matz native pipeline for 2bRAD assembly.

Description	iPyrad ddRAD pipeline		Matz 2bRAD native pipeline	
	Parameter	Setting	Parameter	Setting
Type of input data	*datatype*	ddrad	N/A	N/A
Restriction enzyme overhang	*restriction_overhang*	CATGC	N/A	N/A
Quality filtering	*max_low_qual_bases* *phred_Qscore_offset* *max_barcode_mismatch* *filter_adapters* ^†^ *filter_min_trim_len*	5 33 0 2 35	*‐minQ* (ANGSD) *‐remove_bads* (ANGSD) trim2bRAD_2barcodes_dedup.pl trim2bRAD_2barcodes_dedup.pl *‐m* (cutadapt)	30 1 0 Default parameters 36
Minimum read depth for base calling	*mindepth_statistical* *mindepth_majrule*	5 5	*‐minInd* (ANGSD) *‐postCutoff* (ANGSD)	1 0.95
Maximum allowed cluster depth within samples	*maxdepth*	10,000	N/A	N/A
Percent similarity required to cluster reads into a locus^a^	*clust_threshold* (using vsearch)	0.91	*‐c* (cd‐hit‐est)	0.91
Maximum number of alleles per site in consensus sequences	*max_alleles_consens*	2	Most common read in a cluster is assigned as the reference (cd‐hit‐est)	N/A
Maximum number of N's (uncalled bases) and heterozygotes allowed in consensus (R1, R2)	*max_Ns_consens* *max_Hs_consens*	0, 0 8, 8	No consensus is inferred	N/A
Minimum number of samples required to share a locus in order to be retained in final assembly	*min_samples_locus* ^a^	4	*‐minInd* (ANGSD)	4
Maximum number of SNPs, indels, or heterozygous sites allowed per locus	*max_SNPs_locus* *max_Indels_locus* *max_shared_Hs_locus*	20, 20 8, 8 0.5	N/A bowtie2 bowtie2 (parameter is used to map individual reads to locus; there is no limit to locus‐level heterozygotes)	N/A N/A default parameters (0 + 0.15*read length = 5.4)
Trim raw read edges	*trim_reads* ^a^	5, −25, 0, 0	*‐q* in cutadapt	15,15
Trim locus edges	*trim_loci*	0, 0, 0, 0	N/A	N/A

*Note*: These are the parameters used after testing various clustering threshold values (iPyrad *clust_threshold* parameter). iPyrad was used for the basis of comparison here; parameters are not directly comparable.

^a^ Parameters altered from default settings.

#### 2.1.5 2bRAD assembly

The 2bRAD data were processed separately for each clade using the de novo pipeline developed by Wang et al. (2012) and modified to incorporate deduplication and a triple‐barcoding scheme. This modified pipeline splits reads by in‐read barcode and at the same time deduplicates them based on the ligated degenerate tag. Quality filtering was achieved using cutadapt (https://cutadapt.readthedocs.io/en/stable/#) to remove any tags shorter than the designated 36 bp length, and we once again tested 16 clustering threshold values (0.80–0.95) to assess the effect of this parameter using cd‐hit‐est (Fu et al., 2012), with a “cluster‐derived reference” produced by concatenating the most common representatives of each cluster. This reference was formatted using bowtie2 v.2.3.5.1 (Langmead & Salzberg, 2012) and samtools v.1.9 (Li et al., 2009). The trimmed and filtered reads were mapped to the cluster‐derived reference using bowtie2 with default parameters, and ANGSD v.5.2.3 (Korneliussen et al., 2014) was used to make genotype calls and build consensus sequences. The same missing data threshold was used for the 2bRAD data (*minInd* parameter set to 4 in ANGSD; see Table 3 for further details). A detailed guide to all scripts can be found at https://github.com/z0on/2bRAD_denovo, and details regarding the bioinformatics pipeline for our dataset can be found in Supporting Information.

#### 2.1.6 Consistency between bioinformatics pipelines

As detailed above, we assembled each dataset – 2bRAD and ddRAD – using different pipelines (Matz Lab and iPyrad, respectively) that correspond to the typical user workflow for each type of data. However, to examine how different bioinformatics pipelines may affect assemblies, we ran each dataset through the reciprocal pipeline – 2bRAD data using iPyrad and ddRAD data using the Matz Lab pipeline – and reported general characteristics of the resulting assemblies. All data have been made publicly available (see Supporting Information on Dryad) for further investigation.

To analyze the 2bRAD data with iPyrad, we used the same parameter settings as described above in section 2.1.4, except for changes in the following parameters: data type (*datatype = gbs*), restriction overhang sequence (*restriction overhang* = TGCAG), minimum read length after adapters have been trimmed (*filter_min_trim_length* = 20), and how much to trim raw reads (*trim_reads* = 0,0,0,0). To run the ddRAD data through the Matz Lab pipeline, we began by deduplicating and filtering reads using iPyrad (steps 1 and 2 in iPyrad), followed by the same protocol as described in section 2.1.5, starting at the cluster‐derived reference step (see Supporting Information for a detailed walkthrough and associated output files). No other modifications were made to the Matz Lab pipeline for processing ddRAD data.

To compare the performance of reciprocal bioinformatics pipelines, we examined basic characteristics of final assemblies, including numbers of sites, loci, SNPs, parsimony‐informative sites, average read depth per individual, and proportions of missing data. We calculated the average read depth per individual (across all variable sites in the final assemblies) by extracting the “DP” element from vcf files; this element indicates the read depth for each sample at a given site. We performed this calculation using the *vcfR* package (Knaus & Grunwald, 2017) within a custom R script (see Supporting Information). All subsequent analyses were performed only using the pipelines that corresponded to each data type (i.e., the Matz Lab pipeline for 2bRAD and iPyrad for ddRAD).

### 2.2 Phylogenetic inference

To assess the impact of the type and quantity of data on estimating phylogeny, we estimated phylogenetic trees under maximum likelihood at each sampling depth (*t1*, *t2*, *t3*, and *total*) across both methods and both clades (Figure 2b); this generated 16 trees. Phylogenies were estimated using RAxML‐ng v.0.5.1b (Kozlov et al., 2019; Stamatakis, 2014) with the GTR + Γ model using entire locus sequences in a concatenated matrix, with clades run separately. We used 10 searches to estimate the optimal tree and 200 replicates to calculate bootstrap proportions on the best likelihood tree. We examined bootstrap support and branch lengths using R v.3.6.3 (R Core Team, 2018) with the packages *ape* (Paradis & Schliep, 2019), *phangorn* (Schliep, 2011), and *dplyr* (Wickham et al., 2018), and we visualized data using *cowplot* (Wilke, 2017), *ggplot2* (Wickham, 2016), and *ggtree* (Yu et al., 2017; scripts in Supporting Information).

To quantify the deepest genetic divergence within a clade, we used PAUP* to calculate the (uncorrected) p‐distance between randomly chosen ingroup and outgroup individuals (*A. hahneli* 2a and *E. tricolor* 2; *R. chiricahuensis* 1a and *R. berlandieri* 1a; see Table S1 for sample coding), using the *total* datasets and including variable and constant sites (see Supporting Information). Similarly, we determined the deepest genetic divergence for each clade using sequences (1965 bases) of the *12 S*–*16 S* mitochondrial ribosomal gene from GenBank accessions AY779226 for *R. chiricahuensis*, AY779235 for *R. berlandieri*, HQ290998 for *A. hahneli*, and HQ291001 for *E. tricolor*.

### 2.3 Phylogenetic signal in SNP data

We compared the amounts of phylogenetic signal or information (the converse of homoplasy or noise) in 2bRAD and ddRAD datasets. Although there is no universally accepted measure of phylogenetic information, we considered three measures that discriminate among alternative solutions in the 16 datasets: the number of parsimony‐informative sites (characters), the distribution of unambiguous synapomorphies on a tree, and the retention index (Farris, 1989), all calculated using PAUP* 4.0a, build 166 (Swofford, 2002). For these analyses, we only used variable sites (SNPs).

#### 2.3.1 Parsimony‐informative sites

A parsimony‐informative site is one that does not have the same length (number of steps) on all trees, and so it can be used to discriminate among alternative trees under the parsimony criterion. At least two taxa must have one state, and two others must have a different state, for a character to be parsimony informative. The number of parsimony‐informative sites is reported by PAUP* as standard output.

#### 2.3.2 Unambiguous synapomorphies

We examined the distribution of unambiguous synapomorphies (or more simply, changes) on each branch of the tree. Unambiguous synapomorphies provide clear evidence of branch support because they have only one reconstruction on a tree; in other words, with a synapomorphy, all descendants of a common ancestor share a state which is not present in any other taxa on the tree, and support is unanimous because no other reconstruction of the data is equally parsimonious. In contrast, ambiguous synapomorphies have alternative equally parsimonious reconstructions, and it is not possible to determine on which branch the change occurs (Swofford, 2002). Thus, the number of unambiguous synapomorphies informs us how much unambiguous (under parsimony) phylogenetic information is contained in the data along each branch of the tree. To determine the numbers of unambiguous changes on each branch, we optimized each dataset on its optimal likelihood tree under the accelerated transformation (ACCTRAN) algorithm (Figure 2c) and used custom R scripts to parse the PAUP* output (see Supporting Information).

To compare the phylogenetic information across sampling depths and datasets, we divided the number of unambiguous changes on each branch by the total changes on the tree and plotted the proportions on the branches. For the root edge, however, we plotted the aggregate changes for the two edges descending from the root node in a single graph because it is not possible to determine on which of the two edges the change occurs. For example, if a SNP has state G in the outgroup but A in all ingroup taxa, one cannot determine whether the change between G and A occurred on the branch ancestral to the outgroup or on the branch ancestral to the ingroup.

#### 2.3.3 Retention index

For each dataset, we obtained the retention index from PAUP* by heuristic search using the *hsearch* command. This measure ranges from 0 (no signal) to 1.0 (no homoplasy). The retention index is typically not correlated with the number of characters or taxa, allowing for comparison between datasets of different sizes (Archie, 1996).

### 2.4 Missing data and allelic dropout

#### 2.4.1 Missing data

A common feature of RADseq datasets is variation in missing data, which may bias phylogeny estimation (Crotti et al., 2019; Eaton et al., 2017). We calculated the proportion of missing data (number of matrix cells with “?” or “N,” divided by the total number of cells) in the SNP datasets, for each sampling depth and each individual, using the *missdata* command in PAUP*.

#### 2.4.2 Allelic dropout and phylogenetic signal

Missing data may have several sources, such as poor DNA quality, variation in library preparation, or selection of assembly parameter values. An important biological cause of missing data is allelic dropout, in which a mutation at a restriction site prevents cutting of that fragment so that the putative locus “drops out” of the final assembly; Eaton et al. (2017) referred to this as “mutation‐disruption.” Distinguishing allelic dropout from other causes of missing data can be difficult. We used a phylogenetic criterion to identify allelic dropout by examining the patterns of gains and losses of loci on a tree, under the assumption that close relatives share the same pattern of missing loci (Eaton et al., 2017). In other words, losses showing phylogenetic signal are most likely due to allelic dropout, as opposed to randomly distributed losses of a locus, which might be due to non‐biological causes.

Although our question of allelic dropout is similar to that of Eaton et al. (2017), who used simulated RADseq datasets to investigate the occurrence and patterns of missing data caused by allelic dropout, our approach is different. We first inferred patterns of gains and losses of loci by analyzing the SNP data under Dollo parsimony, which is appropriate for analyzing allelic dropout because it assumes that a locus will be gained only once on the tree, can be lost multiple times, and is not regained if lost (Swofford, 2002). For each assembly, cells with non‐missing nucleotide data were recoded as 1, or “present,” and cells with missing data were recoded as 0, or “absent.” Sites with an alignment gap were excluded (<2% of sites). Using PAUP*, we determined the numbers of unambiguous synapomorphies (changes) on each branch by optimizing each dataset onto its best tree as before (Figure 2c). Allelic dropout was quantified by counting the unambiguous changes from 1 to 0, using R scripts to parse PAUP* output from the command *describe*/*apolist chglist diag*. We then plotted the proportions of changes on each branch for all sampling depths.

Not all instances of dropout are equally informative about phylogeny. A locus that undergoes a single loss on a tree has maximum signal (no homoplasy), but one that shows, for example, four losses on a tree of 10 tips has little signal and is highly homoplastic. To determine whether an instance of allelic dropout has significant signal, we compared its expected number of changes on the tree for each locus (null expectation) to the observed number of changes with a chi‐square test, using the *total* datasets (see Supporting Information for further explanation).

### 2.5 Repeatability

Due to stochasticity in library preparation and sequencing, RADseq methods may not be ideal for augmenting an existing dataset (Andrews et al., 2016). If re‐sequencing a sample yields only a small fraction of the original loci, sequencing more deeply may be required to capture sufficient loci shared across previously and newly sequenced samples. To assess the repeatability of re‐sequencing, a replicate library was constructed and sequenced for two individual frogs from each clade using an ingroup and outgroup species (*R. chiricahuensis* and *R. berlandieri*; *A. hahneli* and *E. anthonyi*) for both 2bRAD and ddRAD. Using custom scripts (see Supporting Information), we quantified repeatability as the number of loci shared by the two replicates divided by the total number of unique loci in both replicates.

### 2.6 Time and cost considerations

The authors who prepared the libraries (E.A.C and R.D.T) had no prior experience with either method and were guided by experienced researchers (see Acknowledgments). We briefly compared the methods qualitatively in terms of overall difficulty relative to standard laboratory techniques and quantitatively in overall cost of library preparation and sequencing, library preparation time, use of specialized equipment, and computational time required for each bioinformatics pipeline. All costs were made based on estimates from 2018.

## 3 RESULTS

### 3.1 Dataset characteristics

Relatively fewer reads were obtained for 2bRAD than requested as compared to ddRAD (Table 1), potentially related to nucleotide base diversity problems with the Illumina HiSeq 4000 chemistry (UT GSAF technical staff, personal communication). When data were analyzed with their respective pipeline, the average read depth per site was 10.10/9.88 for 2bRAD and 21.40/18.10 for ddRAD (*Epipedobates/Rana*, respectively; Table 1). The two methods were consistent in the number of sites recovered for each clade (~3.5 M for *Epipedobates* and ~8.5 M for *Rana*; Table 1). However, 2bRAD recovered 2.8/3.4 times (*Epipedobates/Rana*) more loci and 3.3/2.4 times (*Epipedobates*/*Rana*) fewer SNPs than ddRAD. Interestingly, 2bRAD recovered 8.9/7.8 times fewer parsimony‐informative sites (PIs) and 2.7/3.3 times fewer PIs per SNP than ddRAD, meaning that PI sites were less frequent in 2bRAD data than in ddRAD (*Epipedobates/Rana*). Overall, the differences among the datasets were due primarily to the library preparation, sequencing methods, and analysis pipelines rather than differences in clades. These patterns were observed across all sampling depths (Tables 4 and 5).

**TABLE 4:** Results of assembly pipeline for complete dataset (*total* sampling depth). Rows with bolded text indicate assemblies used for all subsequent analyses.

Dataset	Bioinformatics processing pipeline	Avg. read depth^a^	Missing data (%)^b^	Total sites	Total loci	Total SNPs^c^	Total PIs^d^	SNPs per locus	SNPs per site	PIs per SNP	PIs per locus
***Epipedobates***											
2bRAD	iPyrad	16.2	45.8	2,377,133	76,739	129,433	59,616	1.69	0.05	0.46	0.78
	Matz	9.4	50.6	3,208,050	89,952	63,070	8196	0.70	0.02	0.13	0.09
ddRAD	iPyrad	29.9	56.3	3,558,310	32,371	208,428	73,187	6.44	0.06	0.35	2.26
	Matz	5.5	57.4	2,798,131	30,376	19,583	2320	0.64	0.007	0.12	0.08
***Rana***											
2bRAD	iPyrad	14.6	51.4	4,835,880	156,037	210,491	77,409	1.35	0.04	0.37	0.50
	Matz	8.8	44.3	9,133,414	255,197	161,952	19,281	0.63	0.02	0.12	0.08
ddRAD	iPyrad	26.5	43.6	8,312,261	75,393	381,817	149,816	5.06	0.05	0.39	1.99
	Matz	18.7	34.1	1,558,513	14,196	9428	1016	0.66	0.006	0.11	0.07

*Note*: Native pipelines are in bold.

^a^ Average depth across all individuals.

^b^ Proportion of missing cells in SNP datasets.

^c^ SNPs, single‐nucleotide polymorphisms.

^d^ PIs, parsimony‐informative sites.

**TABLE 5:** Characteristics of data at different sampling depths for 10 individuals, not including replicates.

Sampling depth	ddRAD								2bRAD							
	Sites	Loci	SNPs^a^	PIs^b^	SNPs^a^/ locus	Unamb. synapomorphies	Gains/losses^c^	Informative gains/losses^c^	Sites	Loci	SNPs^a^	PIs^b^	SNPs^a^/locus	Unamb. synapomorphies	Gains/losses^b^	Informative gains/losses^b^
***Rana***																
*t1*	2,255,590	20,486	88,827	25,125	4.34	13,841	210,596	–	5,862,785	164,607	65,154	640	0.40	370	21,780	–
*t2*	4,803,143	43,589	203,795	74,113	4.68	45,327	387,825	–	7,466,955	209,074	108,920	4327	0.52	2666	69,256	–
*t3*	6,846,652	62,100	306,420	120,631	4.93	76,924	528,138	–	8,616,742	240,887	145,448	12,963	0.60	8358	120,121	–
*Total*	8,312,261	75,393	381,817	149,816	5.06	98,161	659,853	328,486	9,133,414	255,197	161,952	19,281	0.63	12,838	143,812	49,416
***Epipedobates***																
*t1*	1,051,484	9561	57,393	20,177	6.00	8823	113,881	–	1,846,516	51,968	27,783	580	0.53	243	62,461	–
*t2*	2,004,556	18,225	114,226	44,429	6.27	19,370	210,225	–	2,652,204	74,401	51,073	4279	0.69	2359	101,682	–
*t3*	2,655,144	24,151	153,665	58,215	6.36	26,135	233,800	–	2,990,659	83,862	59,186	6740	0.71	4082	110,872	–
*Total*	3,558,310	32,371	208,428	73,187	6.44	34,858	438,261	325,269	3,208,050	89,952	63,070	8196	0.70	5154	114,943	85,950

^a^ Single‐nucleotide polymorphisms.

^b^ Parsimony‐informative SNPs.

^c^ Calculated from binary datasets.

**FIGURE 4:**
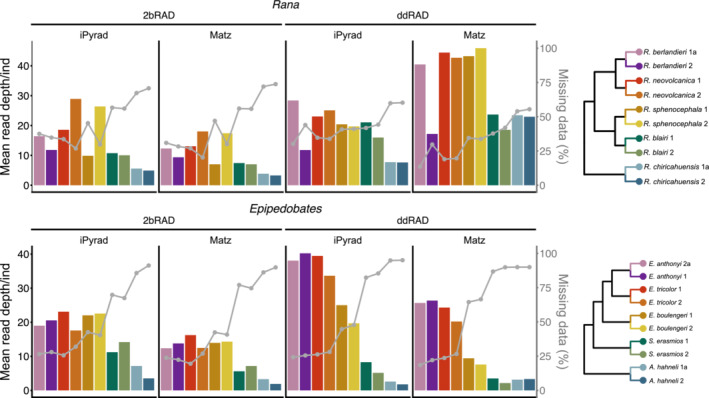
The distribution of read depth (average number of reads per individual per locus) in *total* SNP datasets, including from reciprocal pipelines (left axis, in black), compared to mean proportions of missing data for *total* SNP datasets (right axis; in gray).

After using reciprocal bioinformatics pipelines to process datasets, we found that the pipelines typically used for each data type (i.e., Matz Lab pipeline for 2bRAD data and iPyrad for ddRAD data) recovered more total sites and loci for that data type (Table 4). However, iPyrad consistently recovered a greater number of SNPs (and correspondingly, PIs) for both 2bRAD and ddRAD datasets than the Matz Lab pipeline. In some cases, the discrepancy between SNPs recovered using iPyrad and Matz Lab pipeline was striking; for example, in *Epipedobates* ddRAD dataset, iPyrad recovered 208,428 SNPs, as compared to 19,583 recovered using the Matz Lab pipeline, an increase of more than 10‐fold. Proportions of missing data were comparable for datasets regardless of which bioinformatics pipeline was used, although average read depth per individual was consistently lower in the data processed using the Matz Lab pipeline (Figure 4).

### 3.2 Phylogenetic inference

**FIGURE 5:**
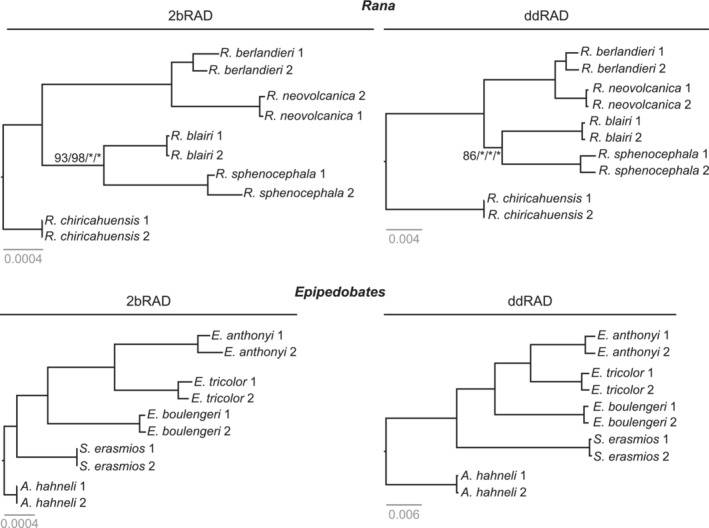
*Rana* and *Epipedobates* maximum likelihood trees for the *total* sampling depth datasets; replicate samples were pruned. Only bootstrap values <100% are shown. All trees reconstructed from remaining sampling depths had node support >0.85. See Table S1 for sample codes.

Maximum likelihood analyses of the *Rana* and *Epipedobates* datasets at all sampling depths and across methods yielded the same topology for each clade (Figure 5). The *Epipedobates* tree showed the same relationships found by Santos et al. (2009) and Tarvin et al. (2017). However, the *Rana* tree differed from recently published trees. Although previous studies found *R. blairi* to be the sister species of *R. berlandieri* + *R. neovolcanica* (Hillis & Wilcox, 2005; Yuan et al., 2016), we found *R. blairi* to be the sister species of *R. sphenocephala*. Bootstrap support values were 100% across nearly all nodes on trees, regardless of taxon, method, or sampling depth, with just a few exceptions (Figure 5).

Interestingly, the relative branch lengths differed between the 2bRAD and ddRAD trees. In the *Epipedobates* and *Rana* ddRAD trees, the tips of the ingroup taxa were roughly the same distance from the root (Figure 5, right column), similar to an ultrametric tree. In contrast, in the 2bRAD trees, the ingroup tips were at varying distances from the root, and this was more pronounced in *Epipedobates* (Figure 5).

For all datasets, the amount of sequence divergence between ingroup and outgroup was greater for *Epipedobates* than for *Rana*. For the 2bRAD data, the p‐distance between the ingroup and outgroup was 0.02297 for *Rana* and 0.03429 for *Epipedobates* (*Epipedobates* is 1.49× larger). For the ddRAD data, the p‐distance was 0.03059 for *Rana* and 0.03931 for *Epipedobates* (1.29× larger). For the *12 S*–*16 S* sequences, the p‐distance was 0.0880 for *Rana* and 0.1390 for *Epipedobates* (1.58× larger).

### 3.3 Phylogenetic signal in SNP data

#### 3.3.1 Parsimony‐informative sites

**FIGURE 6:**
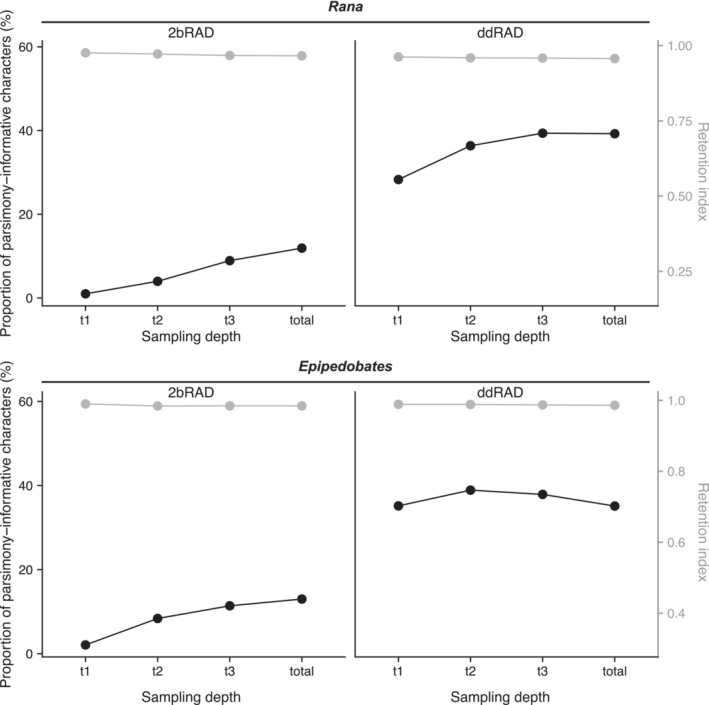
The proportion of parsimony‐informative sites to total sites (left axis; in black) and retention indices (right axis; in gray) between RAxML trees from SNP datasets with four different sampling depths.

The number of PIs was much higher in ddRAD than in 2bRAD within each taxon, even though the total number of sites was similar. Notably, in both clades, the PIs/SNP and PIs/locus ratios were much higher in ddRAD than in 2bRAD, with ddRAD having about three times as many PIs/SNP and 25 times as many PIs/locus than 2bRAD (Table 1). However, the lower PIs/SNP ratio in 2bRAD data may be partly attributable to differences in pipelines, as iPyrad recovered approximately three times more PIs/SNP than the Matz Lab pipeline in both data types; PIs per locus remained low in 2bRAD for both pipelines (Table 4). Using native pipelines, the PIs/SNP ratio increased continuously with sampling depth in 2bRAD datasets, suggesting that coverage limited locus inference in 2bRAD. In contrast, in the ddRAD datasets, the PIs/SNP ratio reached a plateau at *t2* or *t3* and decreased slightly in *total* in both clades (Figure 6).

#### 3.3.2 Unambiguous synapomorphies

We describe the distribution of unambiguous synapomorphies on branches at four levels: the root branch or edge, the intermediate branches (in *Rana*, the two sister branches descending from the ingroup ancestral node, and in *Epipedobates*, the two sequential branches descending from the ingroup ancestral node), the shallow branches (those that are ancestral to the pair of tips comprising a species), and the tip branches (those with no descendants).

**FIGURE 7:**
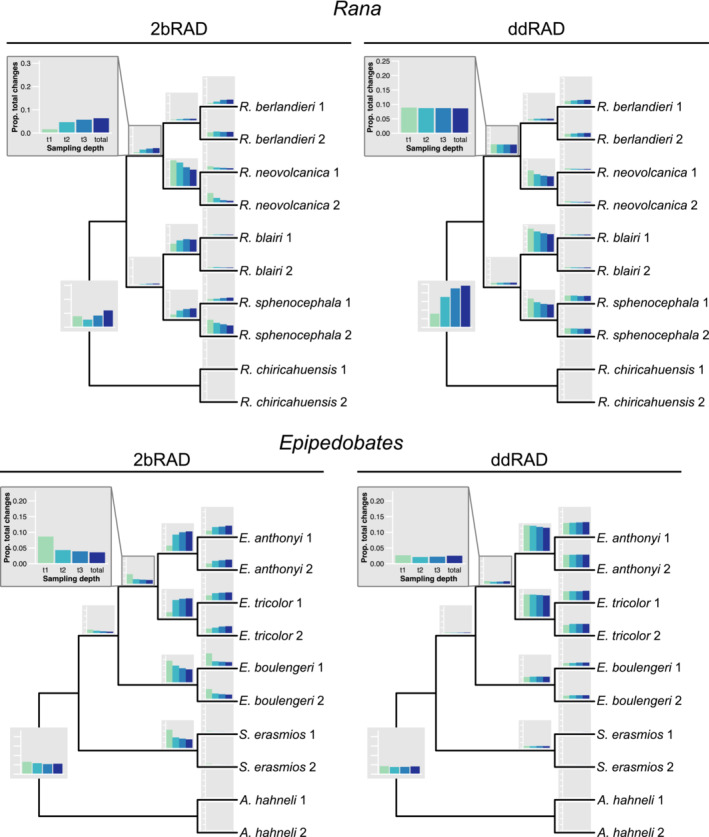
The proportion of unambiguous changes to the total number of SNPs along each branch of the *Rana* and *Epipedobates* trees for each sampling depth, calculated using SNP datasets. This metric provides an estimate of the amount of phylogenetic information.

Proportions of unambiguous synapomorphies on branches (or more simply, changes) were generally similar between 2bRAD and ddRAD (Figure 7 and Figure S1), although proportions of changes in ddRAD were not affected by sampling depth as much as in 2bRAD. With the exception of the *Rana* ddRAD dataset, root edges had relatively fewer changes than the ingroup branches overall. Relatively few changes were found on the intermediate branches in both clades. In both 2bRAD and ddRAD, the shallow branches generally had the largest proportions of changes. The tip branches, not surprisingly, had low proportions of changes, and these were typically higher in the recently diverged species and higher in *Epipedobates* than in *Rana*. Tip branches of the outgroup species (*A. hahneli* and *R. chiricahuensis*) had fewer changes than the ingroup branches (Figure 7 and Figure S1).

#### 3.3.3 Retention index

Retention indices were very high in all analyses and did not vary substantially between 2bRAD and ddRAD datasets (Figure 6). We noted a slight decrease in retention indices with increasing sampling depth.

### 3.4 Missing data and allelic dropout

#### 3.4.1 Missing data

**FIGURE 8:**
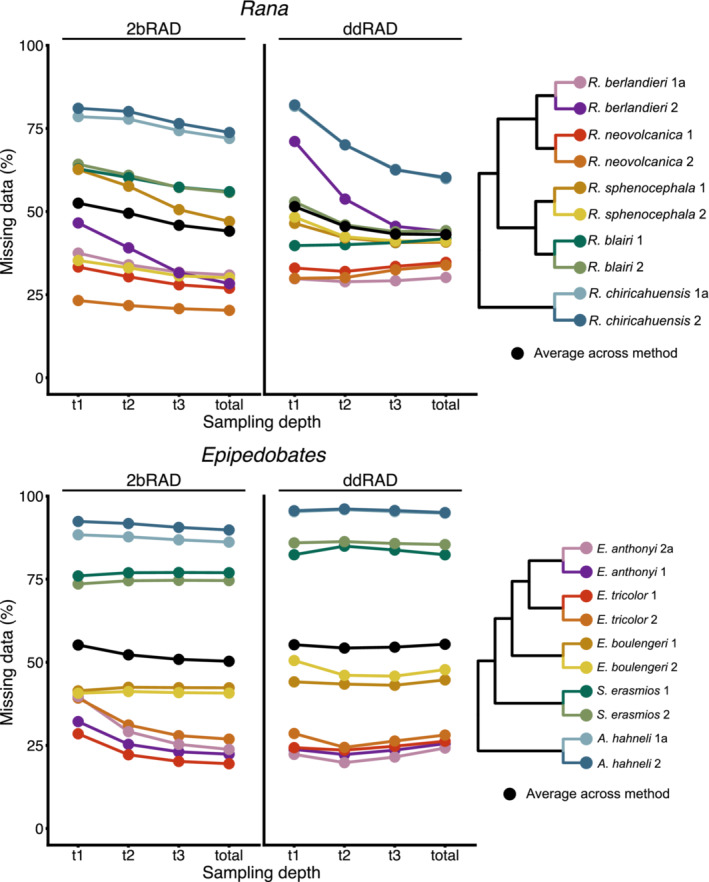
Proportions of missing data contained in each SNP dataset at varying sampling depths per individual and averaged across taxa. For samples in which two replicates were sequenced, only replicate “a” (see Table S1 for coding) was included here.

The proportion of matrix cells with missing data was comparable between RADseq methods (including when assembling with reciprocal pipelines) and across sampling depths (Figure 8; Table 1). The distribution of missing data among individuals varied widely in that recently diverged species (*E. anthonyi*, *E. tricolor*, *R. berlandieri*, and *R. neovolcanica*) had the lowest proportions of missing data across all sampling depths and both methods, ranging from 24.21% to 43.98% in the *total* dataset, while the outgroup species (*S. erasmios*, *A. hahneli*, *R. blairi*, and *R. chiricahuensis*) had the highest proportions, ranging from 41.82% to 94.84% in the *total* datasets (Figure 8). Replicate samples did not contain similar levels of missing data across sampling depths, with differences in missing data proportions between replicates ranging from 0% to 15.3% in ddRAD and from 2.7% to 14.6% in 2bRAD (Table S2). The most similar proportions of missing data between replicates were consistently observed in the *total* sampling depth datasets and least similar in *t1* sampling depth for both ddRAD and 2bRAD. Correspondingly, patterns of missing data were somewhat consistent with patterns of mean read depth per sample (Table 1), in which the most divergent species also had the lowest average read depths and those within the ingroup had the highest, although this relationship was more apparent within ddRAD datasets (Figure 4).

#### 3.4.2 Allelic dropout and phylogenetic signal

**FIGURE 9:**
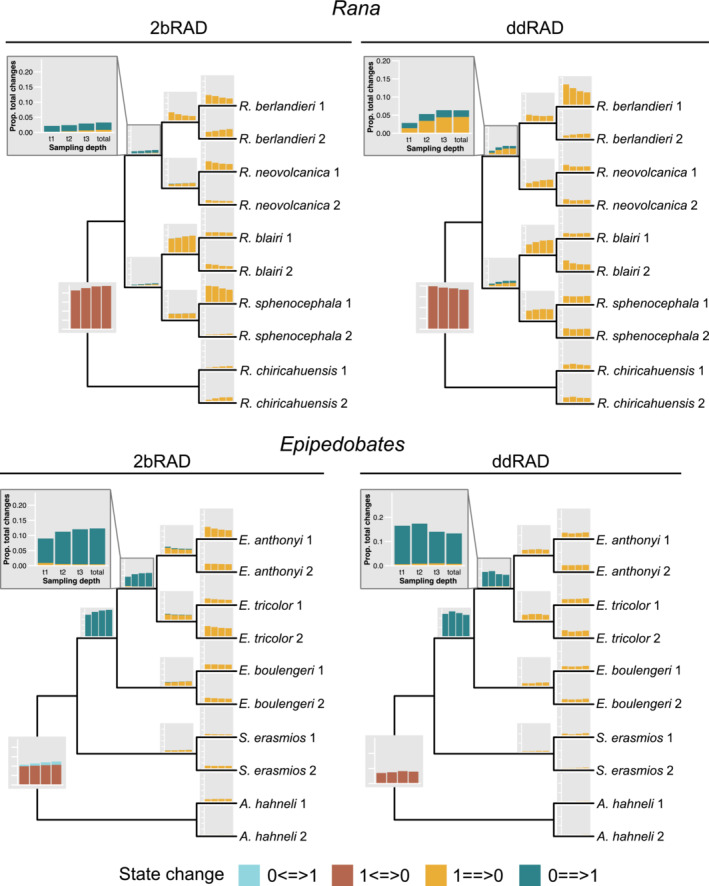
The proportion of unambiguous changes to total number of SNPs along each branch of the *Rana* and *Epipedobates* trees for each sampling depth, calculated using the binary (presence or absence) SNP datasets under Dollo parsimony. Unambiguous changes are categorized based on the type of change; the direction of change along the root edge (double‐headed arrows <==>) is ingroup (first state) to outgroup (second state).

**FIGURE 10:**
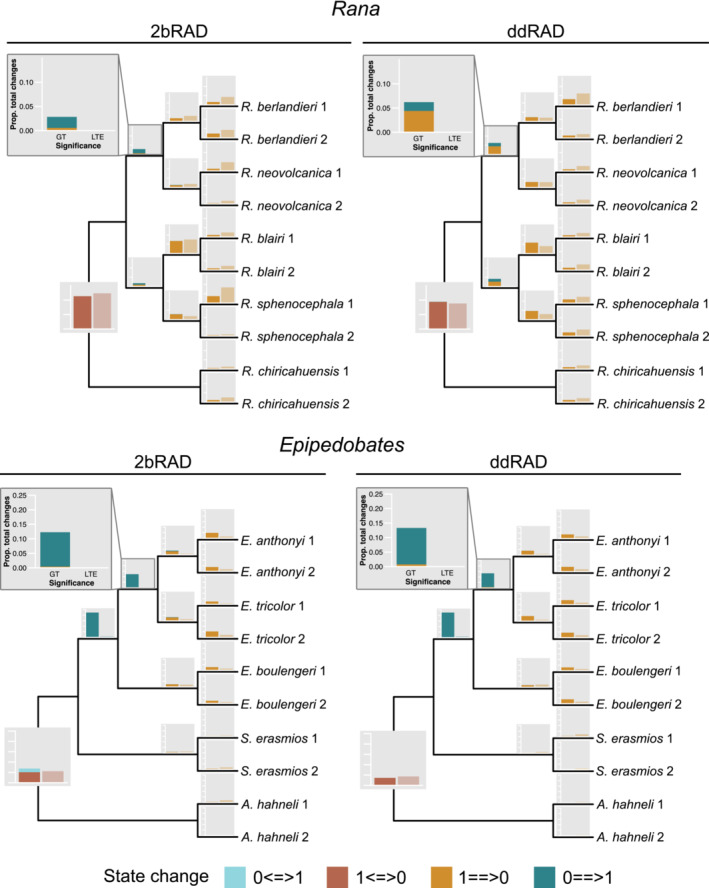
Proportions of unambiguous changes for total datasets from the binary SNP datasets under Dollo parsimony. State changes are categorized based on whether changes were greater than (GT) or less than (or equal to; LTE) expected at random, determined using a chi‐square test. GT changes represent those that exhibited phylogenetic signal and can, therefore, be attributed to allelic dropout. The direction of change along the root edge (double‐headed arrows <==>) is ingroup (first state) to outgroup (second state); thus, 0 <=>1 is a state of 0 in the ingroup and a state of 1 in the outgroup, and single‐headed arrows (==>) are state changes along the remaining branches.

We plotted the relative proportions of allele gains and losses at all sampling depths on the trees (Figure 9). Because the patterns from these analyses did not vary with sampling depth, we only report the results for the *total* dataset (Figure 10). In all datasets, the number of changes (gains or losses) occurring only once on the tree far exceeded the proportions expected under a null model (compare blue bars to orange bars for the first column in each subplot in Figure S2). The exception was the extreme condition of state‐frequency pattern 0011111111, in which the frequency of two changes (no signal) was far fewer than expected by chance. Our overall conclusion from the chi‐square analysis is that both gains of loci and losses of loci (allelic dropout) show overwhelming phylogenetic signal (Table S3).

The patterns of gains and losses on branches differed more between taxa than between sequencing methods, potentially because of topological differences. In both clades, the outgroup species showed very large proportions of allele absence (state 0, typically >70%) as reflected in the amount of missing data (Figure 8); these patterns were generally similar across the sampling depths.

A large proportion of changes (~20%) between 0 and 1 (in either direction) occurred along the root edge in *Rana*; the proportions on the *Epipedobates* root edge were smaller (~10%; Figure 10). In both clades, the proportion of changes having signal was similar to that without signal (compare dark and light brown bars). The changes were largely from 0 (outgroup) to 1 (ingroup), but without information from closest relatives of these clades, we cannot definitively determine whether 0s represents dropout or ancestral absence.

### 3.5 Repeatability

**FIGURE 11:**
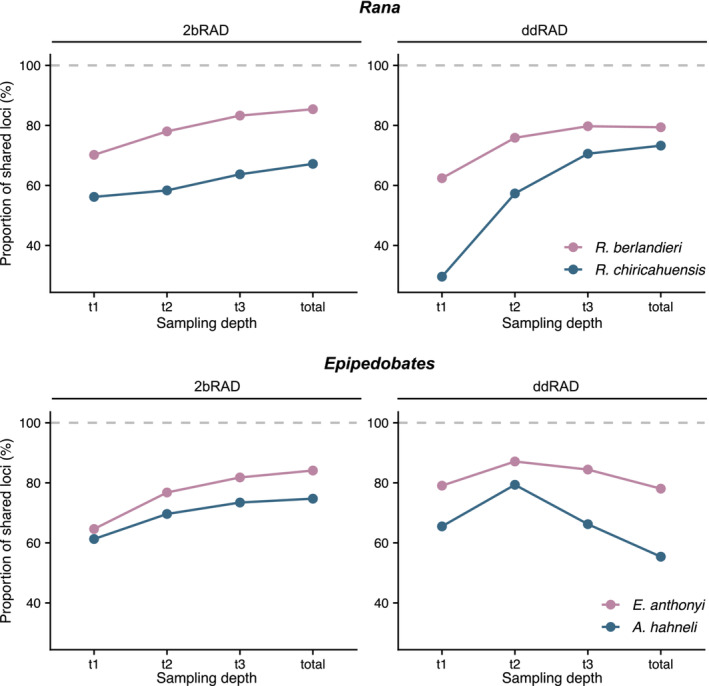
Repeatability of ddRAD and 2bRAD libraries and sequencing. Two replicate samples per dataset were used to assess how many loci were shared between replicates, measured as a proportion of the total number of unique loci in both samples.

Overall, the repeatability of libraries and sequencing was slightly lower in ddRAD than in 2bRAD (Table S2, Figure 11), with an average of 87.93% shared loci recovered between replicates for 2bRAD compared to 83.07% in ddRAD for *total* datasets. Replicates for the outgroups (*R. chiricahuensis* and *A. hahneli*) shared fewer loci than those of ingroup species (*R. berlandieri* and *E. anthonyi*). As sampling depth increased, the proportions of shared loci increased in all samples except for the *t2* dataset for *Epipedobates* ddRAD.

### 3.6 Comparisons of effort, cost, and bioinformatics skills

#### 3.6.1 Laboratory effort

By necessity, our comparisons of person‐effort and cost are qualitative. In our experience, the library preparation for ddRAD is more complex than for 2bRAD in part because it requires selection of appropriate enzymes, specialized reagents such as magnetic beads (e.g., AMPure or SeraPure and Dynabeads), and additional protocols such as size selection using a Pippin Prep. Therefore, ddRAD library preparation took more time, required access to and experience using specialized equipment, potentially making it difficult for inexperienced researchers or labs with less equipment (Table 6). In contrast, we found the 2bRAD library preparation protocol to be more straightforward, involving only a series of PCR steps (see Supporting Information), one enzyme, and no size selection.

**TABLE 6:** Comparison of 2bRAD and ddRAD methods.

	2bRAD	ddRAD
Overall costs^a^	Low	Moderate
DNA required	100 ng	200–500 ng
Laboratory time required	1.5 days	3 days
Library preparation difficulty^b^	Easy	Difficult
Specialized equipment	None	Pippin Prep
High‐performance computer access	Not required	Required
Computational time for bioinformatics assembly	2–4 h^c^	>48 h^c^
Reproducibility	79% (67–85%)^d^	76% (55–79%)^d^
PIs^e^ for each SNP^f^ obtained^g^	0.12	0.37
Cost per SNP^f^	$0.0009	$0.0002
Cost per PI^e^	$0.007	$0.0005

^a^ This is the combined cost for library preparation and sequencing on the Illumina HiSeq 4000.

^b^ This assumes that personnel have no previous experience with library preparation.

^c^ Time required for running full bioinformatics assembly on a high‐performance computer (using iPyrad [Eaton, 2014] for ddRAD data and the Matz Lab pipeline for 2bRAD data). These time estimates are based on our datasets; they are contingent on the amount of data and number of samples a researcher needs to process.

^d^ This is the median and range of the proportion of shared loci recovered for two replicate samples from *total* datasets (Table S2).

^e^ Parsimony‐informative sites.

^f^ Single‐nucleotide polymorphism.

^g^ Value averaged across taxa; see Table 7 and calculations in Supporting Information.

#### 3.6.2 Costs

Library preparation for 2bRAD (including a Bioanalyzer quality check) was less expensive than ddRAD ($11.04/$12.89 for 2bRAD and ddRAD, respectively). Although both 2bRAD and ddRAD protocols require the up‐front purchase of adaptors, ddRAD was particularly costly because it requires relatively more expensive adaptors (see Supporting Information for additional details). Sequencing costs were lower for ddRAD ($40.00 compared to $70.50 for 2bRAD; Table 7) because the higher number of independent loci predicted for 2bRAD required more reads (on average across clades, 14.1 M reads/sample for 2bRAD vs. 6.3 M reads/sample for ddRAD; Table 1). Costs per SNP and PI were around three‐ to five‐fold greater in 2bRAD than in ddRAD (Table 7). Similarly, the cost per unlinked SNP and PI (one per locus) were much more variable but were overall higher in 2bRAD with one exception: the cost per unlinked SNP was approximately 20% cheaper in *Epipedobates*. At more typical levels of sequencing (1–2 M reads/sample), costs would be substantially lower for 2bRAD (50‐bp single‐end reads) than for ddRAD (typically 150‐bp paired‐end reads), although this may result in fewer loci and SNPs retained in the final 2bRAD assemblies (see Supporting Information). To reduce costs while ensuring adequate numbers of SNPs and loci are obtained in 2bRAD assemblies, selective‐base ligation can be performed, in which researchers can more accurately select loci that will be sequenced, which may be particularly useful in species with large genomes (Barbanti et al., 2020). Alternatively, users may wish to use the iPyrad pipeline for 2bRAD data, which produced more SNPs/locus and may make the method more cost‐effective. In the long term, sequencing costs per read will likely remain lower for 2bRAD given its shorter fragment length.

**TABLE 7:** Summary statistics of data and costs for obtaining phylogenetically informative characters from each method.

	2bRAD		ddRAD	
	*Rana*	*Epipedobates*	*Rana*	*Epipedobates*
Sites per sample^a^	9,133,414	3,208,050	8,312,261	3,558,310
Total loci	75,393	89,952	255,197	32,371
SNPs^b^	161,952	63,070	381,817	208,428
PIs^c^	19,281	8196	149,816	73,187
PIs^c^ per SNP^b^	0.12	0.13	0.39	0.35
PIs^c^ per locus	0.08	0.09	1.99	2.26
SNPs^b^ per locus	0.63	0.70	5.06	6.44
Cost of library prep. and lab costs (per sample)^d^	$11.04	$11.04	$12.89	$12.89
Cost of sequencing (per sample)^d^ ^,^ ^e^	$70.50	$70.50	$40.00	$40.00
Cost per sample^d^	$81.54	$81.54	$52.89	$52.89
Cost per SNP^b^	$0.00050	$0.0013	$0.00014	$0.00025
Cost per PI^c^	$0.0042	$0.0099	$0.00035	$0.00072
Cost per SNP/locus^f^	$0.0017	$0.0013	$0.00021	$0.0016
Cost per PI/locus^g^	$0.014	$0.010	$0.00021	$0.0016

^a^ Value averaged across all 12 individuals.

^b^ Single‐nucleotide polymorphisms.

^c^ Parsimony‐informative sites.

^d^ Values obtained using estimates provided in Cost Breakdown section of Supporting Information.

^e^ Estimated costs for sequencing 1.5 M reads per sample.

^f^ If SNPs per locus <1, calculated as: cost per sample divided by (SNPs per locus × total loci). If SNPs per locus >1, calculated as: cost per sample divided by total loci.

^g^ If PIs per locus <1, calculated as: cost per sample divided by (PIs per locus × total loci). If PIs per locus >1, calculated as: cost per sample divided by total loci.

#### 3.6.3 Computational time and bioinformatics skills

Given our experience, the computational time and bioinformatics skills required for ddRAD assembly exceeded those for 2bRAD, though this may depend on researchers' own personal experience level with programming languages and command‐line software. For ddRAD, a maximum runtime of 48 h was allocated for analysis of each sampling depth, using a large memory node (512GB, 32 cores/node). For the *t3* and *total* depths in *Rana* and *Epipedobates*, jobs exceeded the 48‐h limit because of the computationally costly process of within‐sample clustering (iPyrad, Step 3). To remedy this, we submitted a separate job for each sample and then merged samples and ran the remaining assembly steps in iPyrad (Steps 4–7). Thus, iPyrad and ddRAD data require computational resources that are often only available on large computing clusters. By comparison, our 2bRAD analyses at all sampling depths were run on a high‐performance computer within 2–4 h (Table 6).

## 4 DISCUSSION

Much attention has been devoted to exploring the implications of selecting different reduced‐representation genome sequencing methods (Andrews et al., 2016; Cammen et al., 2016; Matz, 2018; McKain et al., 2018). In selecting a method, researchers must weigh the costs of sequencing against the informativeness of the resulting datasets, as well as the equipment and computational resources required to produce and analyze such datasets. These considerations are especially relevant for non‐model organisms or those with large genomes, in which the lack of a reference genome makes assembly challenging.

### 4.1 Sequencing and assembly

In this study, we used two methods (ddRAD and 2bRAD) and targeted a sequencing depth that would yield comparable numbers of sites for each method; indeed, after processing, the total number of sites retained in the *total* datasets was comparable between both methods within each clade (~3.5 M for *Epipedobates* and ~8.5 M for *Rana*). However, each method differed in the quality of data obtained. For example, 2bRAD produced roughly three times more loci than ddRAD (Table 1) yet fewer SNPs and fewer PIs sites per locus, likely attributed to shorter fragment lengths, lower depth of coverage, and pipeline characteristics specific to 2bRAD and the Matz Lab pipeline (Table 4). Up to 38% fewer reads than requested were obtained for 2bRAD, which likely contributed to our ability to assemble loci and confidently infer variants. In both datasets, the numbers of recovered SNPs and loci increased predictably with sampling depth, though patterns in missing data remained the same (Table 5 and Figure 8). Repeatability was similar between methods, although 2bRAD repeatability was less affected by sampling depth (Figure 11).

Each pipeline produced higher numbers of sites and loci for its corresponding data type (i.e., iPyrad for ddRAD and the Matz Lab pipeline for 2bRAD data). However, iPyrad consistently recovered more SNPs and PIs for both datasets than did the Matz Lab pipeline. While the goal of our manuscript was not to compare the two pipelines (i.e., iPyrad vs. Matz Lab pipeline), we suspect that differences in how loci are clustered by each pipeline may have influenced the number of loci obtained (see Table 3 for some relevant parameter comparisons). For example, iPyrad first clusters reads separately within each sample using *vsearch* and a percent similarity cutoff. Then, the most common allele from each locus is clustered among samples, again using *vsearch* and a percent similarity cutoff. In contrast, the Matz Lab pipeline first creates a pseudogenome by clustering reads from all samples using cd‐hit‐est and a percent similarity value. Then, reads from each sample are mapped to the pseudogenome with bowtie2 which uses a minimum score threshold rather than a percent similarity metric. Other differences between pipelines (e.g., approaches to statistical base calling in ANGSD compared to iPyrad) likely also affected the resulting assemblies; future analyses could use the data herein to further explore the implications of differing bioinformatics pipelines for RADseq datasets.

An important consideration for some researchers may be deciding whether to obtain single‐end versus paired‐end reads. To retain consistency across the two methods, we only included results from single‐end read sequencing data for ddRAD, although typical ddRAD workflows involve paired‐end sequencing. Obtaining paired‐end reads may be advantageous for researchers in that it would produce greater numbers of SNPs and PIs, although with diminishing returns if users require unlinked SNPs (Rochette et al., 2017).

### 4.2 Phylogenetic inference

All analyses of the various datasets yielded identical tree topologies for each clade, and bootstrap support was very high, even at the lowest sampling depth. Although the *Epipedobates* topology was the same as recovered by previous studies, the *Rana* topology was not. Hillis and Wilcox (2005) and Yuan et al. (2016) found *R. blairi* to be more closely related to *R. berlandieri* than to *R. sphenocephala*; however, we recovered *R. blairi* as the sister species of *R. sphenocephala*. This difference is likely due to the influence of the mitochondrial genes; Hillis and Wilcox (2005) analyzed only mtDNA, and although Yuan et al. (2016) analyzed both mtDNA and nDNA, their sample of *R. blairi* was represented only by mitochondrial genes. Interestingly, two earlier studies based on allozymes (Hillis et al., 1983) and restriction sites from nuclear rDNA (Hillis & Davis, 1986) also found *R. blairi* to be more closely related to *R. sphenocephala* than to *R. berlandieri*. Thus, it seems that the discrepancy in the position of *R. blairi* is an example of mitonuclear discordance. We consider the SNP phylogeny to be a better estimate of the species trees than mtDNA phylogeny alone.

### 4.3 Phylogenetic signal in SNP data

Overall, the 2bRAD and ddRAD datasets for both clades contained large amounts of phylogenetic signal as measured by numbers of parsimony‐informative characters, retention indices, numbers of unambiguous synapomorphies, and high bootstrap support values (Tables 1 and 5; Figures 4, 6, and 7). This was particularly impressive within the 2bRAD dataset, which was phylogenetically robust to the relatively low proportions of SNPs and PIs compared to the ddRAD datasets. Given that both methods putatively sample the genome randomly, we expected to find roughly the same amount of potential phylogenetic information in the data, yet we recovered more PIs/SNP in ddRAD than in 2bRAD data (Table 1). However, the number of PIs/SNP was similar if the same pipeline was used across both data types (Table 4).

Properties of the enzyme cut sites that differ between methods, differences between clustering algorithms used by iPyrad versus the Matz Lab pipeline, or the lower depth coverage in 2bRAD datasets may have influenced these patterns. For example, the fragments cut by the *Bcg*I enzyme may be more conservative because of the structure of the *Bcg*I cut site (CGA[N]_6_TGC), which requires 6 exact nucleotide matches spaced exactly 6 nucleotides apart, versus that of ddRAD enzyme cut sites, which require two sets of 4–5 exact nucleotide matches but allow up to 50‐nt differences in the number of nucleotides between two cut sites (e.g., GCATG[N]_270‐325_AATT for *Epipedobates*). Differences in clustering algorithms, as reviewed in the Methods and in Table 4, combined with lower average read depth in 2bRAD, could influence the propensity for different alleles to be clustered, labeled as sequencing errors and removed, or split into separate loci. In 2bRAD specifically, the PIs/SNP ratio increased with greater sampling depth, suggesting that phylogenetic information (or statistical base calling) was limited by read depth. For some methods, researchers can choose to modify library preparation protocol and bioinformatics pipelines to optimize amount and quality of data in final assemblies (e.g., Obiol et al., 2014, for strategies to modify data assembly for phylogenetic inference; McCartney‐Melstad et al., 2019, for using a computational approach to select clustering threshold parameter; Barbanti et al., 2020, for performing selective base ligation for size selection in 2bRAD in organisms with large genome sizes). The extent to which RADseq locus‐building pipelines alter downstream analyses is likely to depend on the taxon and enzymes selected and may not be generalizable (e.g., Casanova et al., 2021; O'Leary et al., 2018; Shafer et al., 2017). Nevertheless, because we did not a priori expect the number of PIs/SNP to differ between 2bRAD and ddRAD loci, and because the PIs/SNP ratios were similar between 2bRAD and ddRAD for each pipeline (Table 4), we suspect that the pipelines drove most of the differences in phylogenetic information rather than characteristics of the loci themselves.

The regional patterns of unambiguous synapomorphies on the trees were generally similar between methods and sampling depths, although the proportions of changes along the root edge were smaller in *Epipedobates* than in *Rana*. Typically, the root edges and the shallow branches had proportionately more changes than did the intermediate branches (Figures 4 and 7). This pattern contrasts with the regional distribution of gains and losses of loci, where the largest proportions of gains occurred along the intermediate branches of *Epipedobates* and *Rana*.

### 4.4 Missing data and allelic dropout

#### 4.4.1 Missing data

Because of the ubiquity of missing data in RADseq datasets, much literature has focused on its effects on phylogenetic estimation (e.g., Attard et al., 2018; Eaton et al., 2017; Huang & Knowles, 2016; Leaché, Banbury, et al., 2015), with a general conclusion being that datasets with high amounts of missing data should be retained to optimize phylogenetic inference (e.g., Jiang et al., 2014). However, the role of missing data as a bearer of signal in RADseq data has rarely been studied (Eaton et al., 2017; Leaché & Oaks, 2017).

Patterns of missing data might be expected to vary depending on the RADseq method (Eaton et al., 2017; Hovmöller et al., 2013), but we did not observe this. 2bRAD and ddRAD yielded datasets with comparable levels of missing data (Table 1 and Figure 8), although there were some differences in proportions of missing data which were largely dependent on the taxon. Importantly, there was greater variation in percentage of missing data among species than among sampling depths, implying that even with deeper sequencing, the amount of missing data will be strongly dictated by the divergence patterns of the taxa (Eaton et al., 2017; Ferrer Obiol et al., 2021; Jiang et al., 2014;  Xi et al., 2016). Correspondingly, this also meant that the increased read depth observed in larger sampling depths did not reduce proportions of missing data, though it did provide more phylogenetic information in terms of numbers of SNPs and PIs.

Patterns in missing data and read depth were also shaped by the parameter defining the minimum number of individuals per locus. Our results suggest that including a minimum number of samples in each divergent clade can limit the total amount of missing data, but also that loci recovered from divergent clades (such as *Ameerega*) may not overlap with ingroup clades, in effect limiting the phylogenetic information at deeper nodes in highly divergent datasets. Similarly, read depth decreased with distance from ingroup, but was overall more consistent across samples in 2bRAD (Figure 4).

#### 4.4.2 Allelic dropout

One of the primary causes for missing data in RADseq may be allelic dropout, in which mutations disrupt a recognition site, such that all descendants no longer share a locus (mutation‐disruption; Eaton et al., 2017). Artifacts of this process are apparent when there is a phylogenetic pattern to missing data, in which closer relatives are more likely to share sites and distant relatives are more likely to have lost them (Gautier et al., 2013).

2bRAD and ddRAD did not differ substantially in amounts of allelic dropout. As with phylogenetic signal and missing data, we found greater differences in allelic dropout between clades than between methods, which may reflect differences in the ages of the taxa. If one assumes that the rate of molecular evolution is similar in both clades, then the paucity of changes across the root edge of *Epipedobates* (compared to *Rana*) in conjunction with gains on intermediate depth branches is consistent with an older age for the *Epipedobates* clade (Figure 10). The limits of effectiveness for RADseq at deeper levels of genetic divergence remain unclear (Collins & Hrbek, 2018; Eaton et al., 2017; Harvey et al., 2016; Rubin et al., 2012). Interestingly, we found that gains of loci showed overwhelming phylogenetic signal, while losses showed a mixture of signal and noise, suggesting that allelic dropout is stochastic and not necessarily a good measure of phylogenetic signal (Figure 10).

### 4.5 Repeatability

Sequencing replicate samples is useful for comparing the repeatability of libraries, as well as for determining the rates of genotyping error (Mastretta‐Yanes et al., 2015). Our results were consistent with our predictions: we assumed that because 2bRAD sequencing amplifies fragments at every occurrence of restriction site, 2bRAD libraries would be more reproducible (Andrews et al., 2016). Generally, we found similar levels of reproducibility for both methods across sampling depths, but 2bRAD tended to have higher proportions of shared loci, especially for outgroup species (Figure 11).

### 4.6 Comparisons of effort, cost, and bioinformatics skills

One aspect of the ddRAD protocol that drove its early and enthusiastic adoption was the relatively low effort required to acquire genome‐wide data as well as publicly accessible documentation and well‐established bioinformatics pipelines. Nevertheless, we found that the ddRAD library preparation and bioinformatic pipelines required more time and expertise than 2bRAD. As with ddRAD, protocols and annotated scripts for 2bRAD are available online, but in contrast to ddRAD, the 2bRAD laboratory techniques are straightforward (based on our personal experience, though this may differ depending on the researcher's own personal laboratory experience) and do not require specialized skills or equipment (Wang et al., 2017; see Supporting Information). Taking these considerations into account, the time and effort required for 2bRAD were less than ddRAD. Overall, although sequencing costs were higher for 2bRAD than for ddRAD, this was outweighed by the relatively lower cost of 2bRAD library preparation and the ease of both laboratory protocols and bioinformatics assembly. However, if maximizing numbers of SNPs and PIs is prioritized by researchers, ddRAD is preferred given that costs are lower on a per‐SNP basis (Table 7). Another alternative might be to use 3RAD (Bayona‐Vásquez et al., 2019), which allows for customizability of recovered loci and is quite cost‐effective.

For some study systems, whole‐genome sequencing (WGS) has become an affordable alternative to RADseq. However, a reference genome is necessary to reliably call SNPs from WGS data, and many non‐model systems, including the species included here, still lack high‐quality references. In our focal clades, per sample costs for library preparation and 10× coverage WGS would be between $200 and $250 per sample (~2–4× higher than RADseq; see Supporting Information). Because of the large genomes of amphibians and other non‐model systems, it may be some time before WGS replaces RADseq or other reduced‐representation methods.

### 4.7 Conclusions

Both ddRAD and 2bRAD methods provided abundant and informative data for phylogenetic inference at shallow and intermediate divergence times in non‐model organisms, and so we recommend that selecting between the methods should be based on other considerations, such as person‐effort, costs, and availability of other resources (Table 6). Despite the lower PIs per SNP proportion we identified in 2bRAD, potential users may be interested in unlinked SNPs, which could be facilitated by the greater number of shorter loci obtained in this method, along with more even read depth across phylogenetic divergence. Nevertheless, the quantity of SNPs and PIs are likely to vary with study design, and the PI per SNP ratio varied widely across our methods and clades. Another important consideration which we observed in both methods – as has been observed in other RADseq studies – was the loss of phylogenetic information and shared sites at deeper nodes of the tree. We observed that there appeared to be a level of mitochondrial sequence divergence beyond which phylogenetic information was lost (~15%); genetic divergence calculated from mitochondrial data may be a relevant benchmark with which researchers can gauge the utility of RADseq.

Although 2bRAD was designed primarily for population genomic studies, and despite concerns that the short fragment lengths may pose problems in assembly for taxa lacking a reference genome or having large genomes, we found that 2bRAD data were as reliable and robust for phylogenetic inference as ddRAD data. Phylogenetic reconstruction and support, overall phylogenetic information, proportions of missing data, and rates of allelic dropout in 2bRAD datasets were comparable to those of ddRAD, even at lower sequencing depths. These findings were contrary to common recommendations against the use of 2bRAD (Andrews et al., 2016; Arnold et al., 2013), highlighting the importance of supplementing conceptual comparisons with empirical tests to obtain reliable comparisons between methods.

## AUTHOR CONTRIBUTIONS

**E. Anne Chambers:** Conceptualization (equal); data curation (equal); formal analysis (equal); investigation (equal); methodology (equal); project administration (equal); visualization (equal); writing – original draft (equal); writing – review and editing (equal). **Rebecca D. Tarvin:** Conceptualization (equal); data curation (equal); formal analysis (equal); funding acquisition (supporting); investigation (equal); methodology (equal); project administration (equal); visualization (equal); writing – original draft (equal); writing – review and editing (equal). **Juan C. Santos:** Investigation (supporting); resources (supporting); writing – review and editing (supporting). **Santiago R. Ron:** Investigation (supporting); resources (supporting); writing – review and editing (supporting). **Mileidy Betancourth‐Cundar:** Investigation (supporting); resources (supporting); writing – review and editing (supporting). **David M. Hillis:** Conceptualization (supporting); funding acquisition (supporting); supervision (supporting); writing – review and editing (supporting). **Mikhail V. Matz:** Conceptualization (supporting); formal analysis (supporting); investigation (supporting); methodology (supporting); supervision (supporting); writing – review and editing (supporting). **David C. Cannatella:** Conceptualization (supporting); formal analysis (supporting); funding acquisition (lead); investigation (supporting); methodology (supporting); supervision (lead); visualization (supporting); writing – original draft (supporting); writing – review and editing (supporting).

## CONFLICT OF INTEREST STATEMENT

The authors declare that they have no conflicts of interest. This publication is based, in part, on work by DCC while serving at the National Science Foundation. Any opinion, findings, and conclusions or recommendations expressed in this material are those of the author(s) and do not necessarily reflect the views of the National Science Foundation or the U.S. government.

## Supporting information


Appendix S1

## Data Availability

Data files, along with code to reproduce all analyses, can be found on the Dryad Digital Repository (https://doi.org/10.5061/dryad.fbg79cnsp) as well as on Github: https://github.com/eachambers/epi_rana_radseq. Raw sequence files have been deposited on Sequence Read Archive (project PRJNA930137).
