# Supplementary material for: 2b or not 2b? 2bRAD is an effective alternative to ddRAD for phylogenomics
Source: Ecol Evol. 2023 Mar 8;13(3):e9842. doi: 10.1002/ece3.9842 (PMC9994478; doi:10.1002/ece3.9842)
Supplement: Supplementary file 1 — Appendix S1 [file ECE3-13-e9842-s001.pdf]

Supplemental Information for:
2b or not 2b? 2bRAD is an effective alternative to ddRAD for phylogenomics

E. Anne Chambers, Rebecca D. Tarvin, Juan C. Santos, Santiago R. Ron, Mileidy Betancourth-Cundar, David M. Hillis, Mikhail V. Matz, & David C. Cannatella

TABLE S1 Details of specimens used in this study.

Sample ID	Field number	Species	Locality	Figure code
QCAZ[†] A53659	TNHCFS[a] 6836	*Epipedobates tricolor*	Echaendia, Ecuador	*E. tricolor* 1
QCAZ[†] A53665	TNHCFS[a] 6842	*Epipedobates tricolor*	Echaendia, Ecuador	*E. tricolor* 2
QCAZ[†] A53680	TNHCFS[a] 6857	*Epipedobates anthonyi*	Pasaje, Ecuador	*E. anthonyi* 1
QCAZ[†] A53682[‡]	TNHCFS[a] 6859	*Epipedobates anthonyi*	Pasaje, Ecuador	*E. anthonyi* 2a, 2b
QCAZ[†] A58309[‡]	RDT[b] 0089	*Ameerega hahneli*	Canelos, Ecuador	*A. hahneli* 2a, 2b
QCAZ[†] A58310	RDT[b] 0090	*Ameerega hahneli*	Canelos, Ecuador	*A. hahneli* 1
ANDES[§]-A2461	RDT[b] 0153	*Epipedobates aff. boulengeri*	Ladrilleros, Colombia	*E. boulengeri* 1
ANDES[§]-A2464	RDT[b] 0156	*Epipedobates aff. boulengeri*	Ladrilleros, Colombia	*E. boulengeri* 2
ANDES[§]-A2466	RDT[b] 0158	*Silverstoneia erasmios*	Buenaventura, Colombia	*S. erasmios* 1
ANDES[§]-A2467	RDT[b] 0159	*Silverstoneia erasmios*	Buenaventura, Colombia	*S. erasmios* 2
TNHC-GDC[¶] 101225	DRD[c] 2864	*Rana blairi*	South Dakota, USA	*R. blairi* 1
TNHC-GDC[¶] 101226	DRD[c] 2865	*Rana blairi*	South Dakota, USA	*R. blairi* 2
TNHC-GDC[¶] 480	JAC[d] 10530	*Rana neovolcanica*	Jalisco, Mexico	*R. neovolcanica* 1
TNHC-GDC[¶] 527	JAC[d] 10534	*Rana neovolcanica*	Jalisco, Mexico	*R. neovolcanica* 2
TNHC-GDC[¶] 1113[‡]	N/A	*Rana berlandieri*	Tamaulipas, Mexico	*R. berlandieri* 1a, 1b
TNHC-GDC[¶] 1114	N/A	*Rana berlandieri*	Tamaulipas, Mexico	*R. berlandieri* 2
TNHC-GDC[¶] 2034[‡]	JSF[e] 1089	*Rana chiricahuensis*	Arizona, USA	*R. chiricahuensis* 1a, 1b
TNHC-GDC[¶] 2049	N/A	*Rana chiricahuensis*	Arizona, USA	*R. chiricahuensis* 2
TNHC-GDC[¶] 25870	WLH[f] 1176	*Rana sphenocephala*	Texas, USA	*R. sphenocephala* 1
TNHC-GDC[¶] 26064	TJL[g] 608	*Rana sphenocephala*	Texas, USA	*R. sphenocephala* 2

[†]Museo de Zoología, Pontificia Universidad Católica del Ecuador.
[‡]Replicated samples.
[§]Museo de Historia Natural at the Universidad de los Andes.
[¶]Genetic Diversity Collection, UT Biodiversity Center.
[a]Texas Natural History Collection Field Series (UT Biodiversity Center).
[b]Rebecca D. Tarvin.
[c]Drew R. Davis.
[d]Jonathan A. Campbell.
[e]John S. Frost.
[f]Wendy L. Hodges.
[g]Travis J. LaDuc.

TABLE S2 Repeatability of RADseq methods. To assess repeatability, we calculated the number of shared loci between replicate samples. The total loci are the number of unique loci (the union) in the assemblies of both replicate samples.

Rana							
		**ddRAD**			**2bRAD**		
**Sampling depth**	**Replicates (two)**	**Shared loci**	**Total loci**	**Missing data (%)†**	**Shared loci**	**Total loci**	**Missing data (%)**
t1	*R. berlandieri*	10,643	17,050	29.8 | 45.1	37,048	52,774	37.5 | 24.6
	R. chiricahuensis	1,401	4,729	81.7 | 75.1	11,267	20,049	78.6 | 73.4
t2	*R. berlandieri*	26,384	34,770	28.9 | 36.4	67,377	86,363	34.0 | 24.8
	R. chiricahuensis	7,836	13,667	70.1 | 64.5	20,077	34,405	77.8 | 72.2
t3	*R. berlandieri*	38,593	48,401	29.2 | 34.7	94,098	112,995	31.8 | 25.9
	R. chiricahuensis	15,529	22,001	62.7 | 58.8	32,797	51,472	74.3 | 67.7
total	*R. berlandieri*	46,217	58,215	30.2 | 35.2	106,541	124,748	30.9 | 26.3
	R. chiricahuensis	20,782	28,364	59.9 | 56.9	41,084	61,144	72.0 | 64.9

Epipedobates							
		**ddRAD**			**2bRAD**		
**Sampling depth**	**Replicates (two)**	**Shared loci**	**Total loci**	**Missing data (%)†**	**Shared loci**	**Total loci**	**Missing data (%)**
t1	*E. anthonyi*	6,673	8,441	22.3 | 26.5	14,748	22,822	39.7 | 25.1
	A. hahneli	279	426	95.3 | 95.6	2,137	3,487	88.4 | 91.4
t2	*E. anthonyi*	13,813	15,857	19.8 | 21.3	33,929	44,189	29.1 | 17.9
	A. hahneli	607	765	95.9 | 96.1	4,528	6,503	87.7 | 90.7
t3	*E. anthonyi*	17,566	20,807	21.5 | 23.5	42,201	51,594	25.3 | 16.2
	A. hahneli	857	1,294	95.3 | 95.4	5,914	8,053	86.8 | 89.6
total	*E. anthonyi*	21,658	27,746	24.2 | 27.0	46,245	54,992	23.8 | 15.7
	A. hahneli	1,166	2,105	94.8 | 94.8	6,755	9,040	86.1 | 88.8

†The pipe symbol separates missing data proportions for replicates a and b, respectively.

TABLE S3 Chi-squared tests of expected vs. observed changes. For each character pattern and each dataset, the total number of permutations for that pattern on the tree is shown (third column), followed on the same line by the expected proportions of changes occurring in each class of changes on the tree (1–5). For each dataset, the observed proportions of changes for each class is shown. P is the probability of accepting the null hypothesis under the chi-squared test (999 permutations). An asterisk following an observed proportion means that a posthoc test indicates that it exceeds the expected by three standard deviations ($p < 0.001$).

**Character pattern**	**Dataset (*total*)**	**Total permutations**	***p*-value (chi-sq)**	**Number of changes of a character on the tree, with proportions of total changes below**				
				**1**	**2**	**3**	**4**	**5**
0000000011	Expected	45		0.111	0	0.178	0.356	0.356
	Rana 2bRAD		<0.001	0.220*	0	0.206	0.140	0.435*
	Rana ddRAD		<0.001	0.394*	0	0.096	0.314	0.197
	Epipedobates 2bRAD		<0.001	0.607*	0	0.246*	0.095	0.052
	Epipedobates ddRAD		<0.001	0.275*	0	0.239*	0.155	0.332
0000000111	Expected	120		0	0.067	0.133	0.267	0.533
	Rana 2bRAD		<0.001	0	0.270*	0.083	0.361*	0.286
	Rana ddRAD		<0.001	0	0.169*	0.423*	0.231	0.178
	Epipedobates 2bRAD		<0.001	0	0.560*	0.263*	0.129	0.048
	Epipedobates ddRAD		<0.001	0	0.543*	0.243*	0.156	0.057
0000001111	Expected	210		0.010	0.019	0.133	0.457	0.381
	Rana 2bRAD		<0.001	0.261*	0.028*	0.144	0.525*	0.042
	Rana ddRAD		<0.001	0.252*	0.156*	0.179*	0.356	0.057
	Epipedobates 2bRAD		<0.001	0.524*	0.203*	0.192*	0.071	0.010
	Epipedobates ddRAD		<0.001	0.369*	0.260*	0.249*	0.098	0.024
0000011111	Expected	252		0	0.048	0.190	0.635	0.127
	Rana 2bRAD		<0.001	0	0.079*	0.754*	0.166	0.001
	Rana ddRAD		<0.001	0	0.179*	0.624*	0.189	0.008
	Epipedobates 2bRAD		<0.001	0	0.760*	0.178	0.060	0.002
	Epipedobates ddRAD		<0.001	0	0.737*	0.169	0.090	0.004
0000111111	Expected	210		0.010	0.038	0.571	0.381	0
	Rana 2bRAD		<0.001	0.047*	0.529*	0.416	0.008	0
	Rana ddRAD		<0.001	0.171*	0.445*	0.354	0.030	0
	Epipedobates 2bRAD		<0.001	0.772*	0.122*	0.100	0.006	0
	Epipedobates ddRAD		<0.001	0.809*	0.103*	0.081	0.006	0
0001111111	Expected	120		0	0.333	0.667	0	0
	Rana 2bRAD		<0.001	0	0.948*	0.052	0	0
	Rana ddRAD		<0.001	0	0.899*	0.101	0	0
	Epipedobates 2bRAD		<0.001	0	0.939*	0.061	0	0
	Epipedobates ddRAD		<0.001	0	0.929*	0.071	0	0
0011111111	Expected	45		0.111	0.889	0	0	0
	Rana 2bRAD		<0.001	0.805*	0.195	0	0	0
	Rana ddRAD		<0.001	0.803*	0.197	0	0	0
	Epipedobates 2bRAD		<0.001	0.894*	0.106	0	0	0
	Epipedobates ddRAD		<0.001	0.942*	0.058	0	0	0

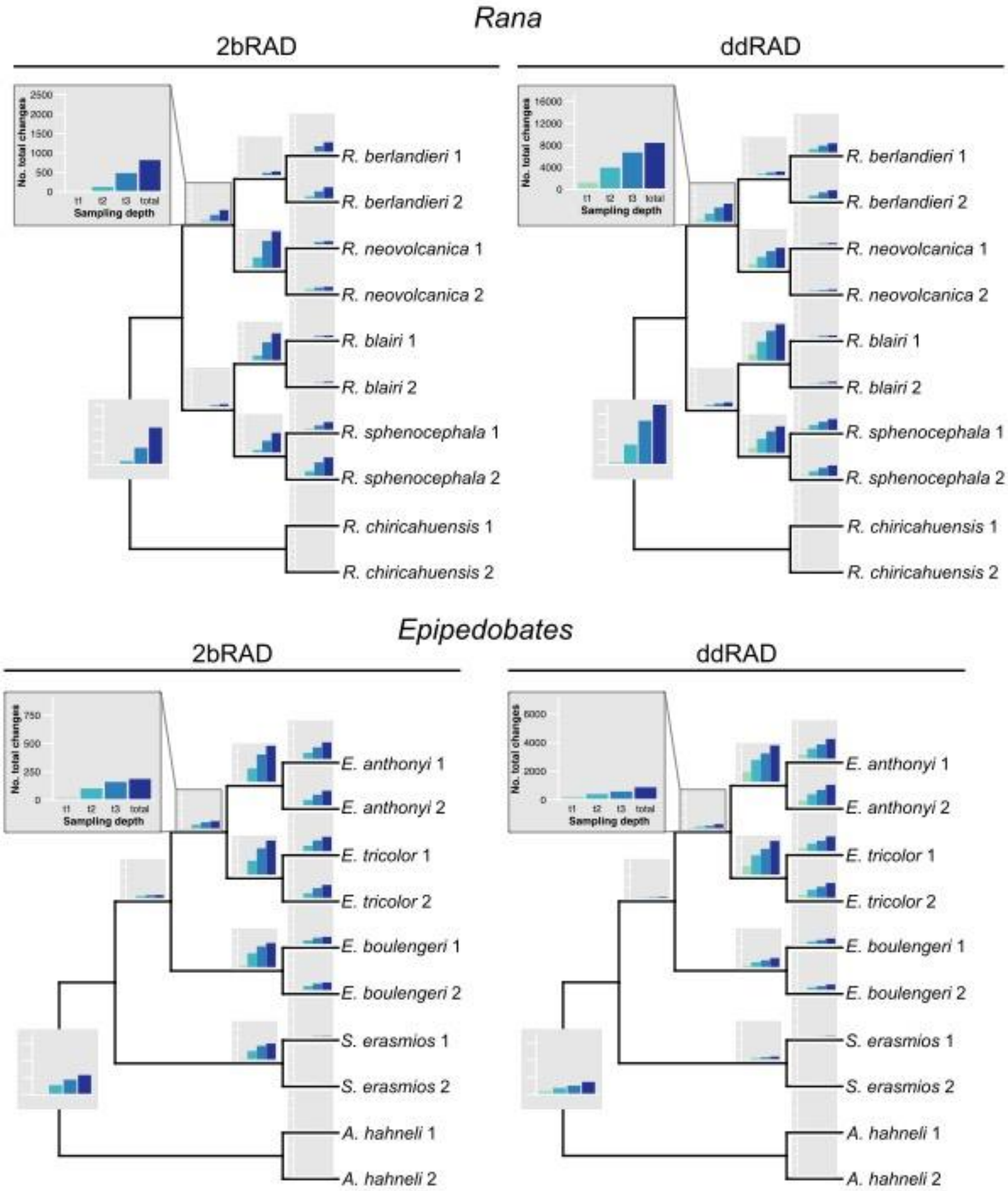

FIGURE S1 The number of unambiguous changes along each branch of the *Rana* and *Epipedobates* trees for each sampling depth, calculated from SNP datasets.

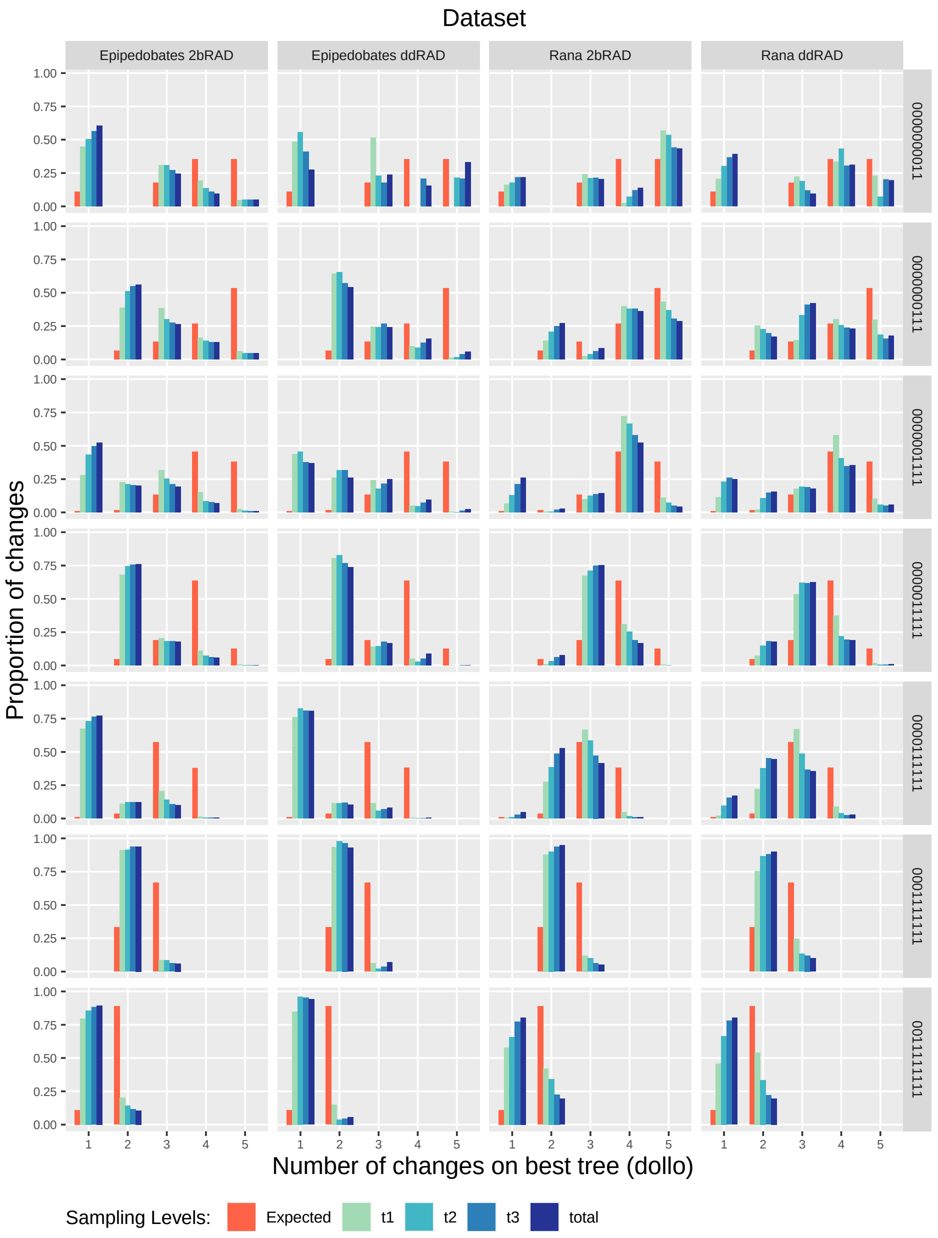

FIGURE S2 Panels comparing the expected and observed character changes for each sampling depth, summarized for each combination of dataset and character state pattern. A goodness-of-fit test (chi-squared) was used to determine whether the distribution of number of changes for a dataset sample (blue bars) was different than expected at random (red bars). Asterisks indicate that the post-hoc comparison between observed and expected is significant at $p < 0.0001$, and n is the total observed changes for the given panel.

CALCULATIONS FOR SEQUENCING

The following equation was used to calculate number of requested reads to be sequenced:
[(Genome size * proportion of genome recovered by enzyme) / average fragment size] * coverage

Sequencing costs at the UT Austin Genomic Sequencing and Analysis Facility (GSAF):
For 2bRAD: 200M reads per lane at a cost of $1,000 per lane (50 bp single-end reads)
For ddRAD: 240M reads per lane at a cost of $1,500 per lane (150 bp single-end reads) or $2,500 per lane (150 bp paired-end reads)

To obtain more realistic sequencing estimates, we calculated the cost for 1.5M reads per sample:
For 2bRAD, (1.5M reads/200M reads)*$1,000 = $7.50 per sample
For ddRAD single-end sequencing, (1.5M reads/240M reads)*$1,500 = $9.38 per sample
For ddRAD paired-end sequencing, (1.5M reads/240M reads)*$2,500 = $15.63 per sample

	Epipedobates	
**Calculated value**	**2bRAD**	**ddRAD**
Estimated genome size	9 Gb	9 Gb
Estimated proportion of genome	0.5%	0.98%†
Average estimated fragment size	36 bp	291 bp†
Depth of sequencing	20X	20X
Number of loci expected	1.25M loci	303,095.7 loci
Minimum reads per sample	25M reads	6.06M reads
Target reads per sample (+20%)	30M reads	7.27M reads
	Rana	
**Calculated value**	**2bRAD**	**ddRAD**
Estimated genome size	6 Gb	6 Gb
Estimated proportion of genome	0.5%	1.21%†
Average estimated fragment size	36 bp	314 bp†
Depth of sequencing	20X	20X
Number of loci expected	833,333 loci	231,210 loci
Minimum reads per sample	16.7M reads	4.62M reads
Target reads per sample (+20%)	20.0M reads	5.55M reads

†Based on results from Agilent Bioanalyzer

REAGENTS REQUIRED

The following are the reagents required for each method. Volumes are for a single reaction; these values were used in Table S5 to calculate costs.

	**2bRAD reagents**	
**Protocol part**	**Reagent**	**Volume**
(a) DNA cleanup		
	Zymo kit	1 kit
(b) DNA single digest		
	NEB buffer	0.8µl
	SAM	0.12µl
	*Bcg*I enzyme	1.1µl
(c) Adaptor priming		
	5Ill-NNRW	3µl
	5Ill-anti NNRW	3µl
(d) Adaptor ligation		
	T4 ligase	1µl
	10X T4 ligase buffer	2µl
	10mM ATP	0.5µl
	Adaptor 5’	1µl
(e) Test PCR		
	P5 10uM	0.2µl
	P7 10uM	0.2µl
	Multiplex 2n 10uM	0.15µl
	Ill-BC91 10uM	0.15µl
	dNTP 2.5uM	2µl
	Phusion Taq	0.2µl
	**ddRAD reagents**	
**Protocol part**	**Reagent**	**Volume**
(a) DNA double digest		
	*Sph*I	0.15µl
	*Mlu*Cl	0.3µl
	Cutsmart buffer	5µl
(b) Adaptor annealing		
	1.1 oligos 40uM stock plate	20µl
	1.2 oligos 40uM stock plate	20µl
(c) Bead DNA cleanup		
	Serapure beads	105µl
(d) DNA quantification		
	Picogreen	
(e) Adaptor ligation		
	P1 adaptor	0.2µl
	P2 adaptor	0.2µl
	T4 ligase	0.625µl
(f) Size selection		

	Pippin Prep cassette	
	Pippin loading solution	10μl
(g) Dynabead cleanup		
	Dynabeads	13μl for pooled samples
(h) PCR and final cleanup		
	PCR1 forward	12.5μl
	P2.4/9	12.5μl
	AccuPrime Taq	1.25μl
	Serapure beads	105μl

COST BREAKDOWN

Costs for all steps associated with our total dataset for 2bRAD and ddRAD protocols presented in this paper. Estimates for sequencing are from the pricing offered in 2017 at the UT Austin Genomic Sequencing and Analysis Facility; in-lab ddRAD costs are taken from Peterson et al. (2012)

Step	2bRAD in-lab cost/sample	ddRAD in-lab cost/sample
(a) DNA preparation		
DNA extraction[a]	$2.50	$2.50
DNA cleaning	$3.00[b]	$0.17[j]
DNA: total	***$5.50***	***$2.67***
(b) Enzyme selection	$0.00	$1.50[k]
(c) Library preparation		
Enzymes	$2.99[c]	$2.64[l]
Oligomers	$0.30[d]	$2.47[m]
Magnetic beads	$0.00	$0.35[n]
Library preparation: total	***$3.29***	***$5.46***
(d) Size selection	$0.00[e]	$0.84[o]
(e) Quality check	$1.25[f]	$1.25[f]
Laboratory costs: subtotal	$10.04	$11.72
Troubleshooting (10%)	$1.00	$1.17
**Laboratory costs: total**	**$11.04**	**$12.89**
(f) Sequencing	$70.50[g]	$40.00[p]
**Total per sample**	**$81.54**	**$52.89[q]**
Laboratory time	1.5 days	3 days
(g) Computational time	2–4 hours[h]	>48 hours[r]
(h) Other considerations	[i]	[s]

Abbreviations: GSAF, UT Austin Genomic Sequencing and Analysis Facility.

[a]Any DNA extraction kit is acceptable; these estimates are based on the Qiagen DNeasy Blood & Tissue Kit ($616 for 250 samples).
[b]This estimate is based on the Zymo Genomic DNA Clean & Concentrator kit ($150 for 50 samples).
[c]This value is the combined cost of the enzymes and reagents used for 2bRAD digestion: BcgI (unit) = $0.27; T4 ligase (unit) = $1.28; Phusion Taq (unit) = $1.44.
[d]This value includes the costs for 2bRAD adaptors and primers (per reaction). Researchers should be advised, however, that 2bRAD adaptors must be purchased in a large batch with a minimal cost of $1,984.
[e]This cost is based on size selection performed using an agarose gel.
[f]This is the cost for two Agilent Bioanalyzer runs at the GSAF (internal pricing).
[g]2bRAD sequencing was performed using the Illumina HiSeq 4000 sequencer with 50 bp single-end reads, which is able to sequence 200M reads/lane at a cost of $1,000/lane. This value was calculated using the average number of per-sample reads obtained for *Rana* and *Epipedobates* (average of 14.1M reads per sample). Thus, sequencing costs were (14.1*1,000)/200 = $70.50 per sample.
[h]All aspects of the 2bRAD bioinformatics assembly can be completed on a personal computer.

[i]The 2bRAD protocol requires no expertise or special equipment.
[j]This value represents the cost of RNAse during extraction; the DNA extraction protocol requires 0.05mg RNAse per sample ($82 for 24mg).
[k]This value is the approximate cost to test three enzyme pairs (enzymes are on average ~$0.50/enzyme pair per reaction; $0.50 x 3 reactions = $1.50).
[l]This value is the combined cost of the enzymes and reagents used for ddRAD digestion: *Sph*I (unit) = $0.20; *Mlu*Cl (unit) = $0.20; T4 ligase (unit) = $0.80; Phusion Taq (unit) = $1.44.
[m]This value is the approximate cost for ddRAD adaptors and oligos for a single reaction, based on the following: P1 adaptors: $1,920 for plate of 96; P2 adaptors: $100 for 12; PCR primers: $250 for 12; P2.2 biotinylated: $200 for 12; stock primers are good for ~1,000 reactions. Researchers should be advised, however, that all oligos must be purchased in a large batch with a minimal cost of $2,470. See Peterson et al. (2012) supplementary materials for additional details.
[n]This value is the combined estimate for two clean-ups using Sera-mag Speedbeads (two clean-ups) = $0.09 (based on estimates provided by Faircloth & Glenn [2011]: https://ethanomics.files.wordpress.com/2012/08/serapure_v2-2.pdf); if using Dynabeads, performing one clean-up = $0.26.
[o]This is the cost for a Pippin Prep 2% cassette; these estimates are taken from the supplementary online documents of Peterson et al. (2012).
[p]ddRAD sequencing was performed using the Illumina HiSeq 4000 technology with 150 bp single-end reads, which is able to sequence 240M reads/lane at a cost of $1,500/lane. This value was calculated using the average number of per-sample reads obtained for *Rana* and *Epipedobates* (average of 6.4M reads per sample). Thus, sequencing costs were (6.4*1,500)/240 = $40.00 per sample. For paired-end reads using the same size selection window and sequencing technology, the cost for 240M reads/lane is $2,500. Thus, sequencing costs for paired-end sequencing would be (6.4*2,500)/240 = $66.67 per sample.
[q]This cost is for sequencing single-end reads; the total per sample cost for 150 bp paired-end reads is the laboratory costs ($68.48) in addition to sequencing costs ($66.67) = $135.15.
[r]For ddRAD single-end reads, the complete iPyrad bioinformatics pipeline took $\geq$48 hours depending on sampling depth; for paired-end reads, we were unable to complete the bioinformatics analysis due time limits. Processing of both single- and paired-end reads requires high-powered cluster computers.
[s]The ddRAD protocol requires experience to operate specialized equipment and perform protocols (e.g., experience using a Pippin Prep machine, performing magnetic bead cleanup using a SPRIPlate).

Whole genome sequencing costs

We also estimated the costs of performing whole genome sequencing (WGS) compared to our 2bRAD and ddRAD cost estimates. We estimated that in-house WGS library preparation costs are approximately $25 per sample. Sequencing costs are based on QB3 Genomics at the University of California Berkeley pricing; WGS costs $2.50 per GB genome size. For 10x coverage, WGS would cost $225 per sample in *Epipedobates* (estimated genome size: 9GB) and $175 per sample in *Rana* (estimated genome size: 6GB). Thus, combined library preparation and sequencing costs amount to $250 and $200 per sample (for *Epipedobates* and *Rana*, respectively).

ALLELIC DROPOUT AND PHYLOGENETIC SIGNAL: ADDITIONAL DETAILS

To derive the null expectation, one must consider the presence/absence of a locus across the ten tips of the tree. For example, if a locus is absent in four tips and present in six (0000111111), there are 210 different ways (permutations) to assign four 0s and six 1s to the ten uniquely labeled tips. For each of these permutations, we optimized the locus on the tree under Dollo parsimony and then calculated the number of evolutionary changes. The frequencies of each category of changes (1, 2, 3, ...) forms the null distribution of possible changes on the tree. In this example, the expected frequency is 2/210 for one change, 8/210 for two, 120/210 for three, and 80/210 for four. Thus, under the null only 0.95% of permutations (2/120) are expected to show perfect fit (one step), while 38.1% (80/210) are expected to show poor fit (four changes, maximum homoplasy).

For each dataset, the characters were divided into state-frequency classes (0011111111, 00011111111, ..., 0000000011) and the null expectation for each was generated by optimizing all unique permutations of states on the tree using the *Permn* function of the R package *DescTools* (Signorell 2020). Each permutation was optimized on the tree, and the number of changes was cumulated for each state-frequency class, similar to permutation tests for phylogenetic signal (Archie 1989).

For each dataset, the expected proportions for each state-frequency class were compared to the observed proportions for 1–5 changes, 5 being the maximum possible given the tree topologies. A chi-squared test was performed, with significance determined by 999 permutations; this yielded 28 chi-squared tests. Because the null hypothesis was (unsurprisingly) rejected at 0.001 in every case, we performed one-tailed post-hoc tests using the chi-squared standardized residuals (observed minus expected divided by the standard deviation of the expected) to determine whether the observed changes significantly exceeded the expected or not for each category (1–5 changes). A stringent critical value of 3.09 standard units (alpha=0.001, one-tailed test) was applied (scripts in Supporting information). For the *total* datasets we used R plots to compare the proportions of observed changes that exceeded the expected signal (i.e., significant signal) to those that did not.

DATA REPRODUCIBILITY

The following schematic details all files contained within Supplemental Information (available here: https://doi.org/10.5061/dryad.fbg79cnsp) required for each analysis. ***N.B.:*** *In most of the raw data and output files,* S. erasmios *samples (RDT0158 and RDT0159) are mislabeled as* S. nubicola. *This was due to a misidentification of the individuals; none of our analyses were affected by this change.*

Chambersetal_SuppMats_Software (code and input data files for visualization)

Directory	Description
**1_Lab_protocols**	
2bRAD_protocol.pdf	Adapted from https://github.com/z0on/2bRAD_GATK *(ddRAD protocol directly from Peterson et al. [2012] Supplementary Materials[1])*
**2_Bioinformatics**	
iPyrad_ddRAD	
scripts/	
extract_data.ipynb	Code to concatenate iPyrad stats files
Matz_2bRAD/	2bRAD bioinformatics pipeline files using Matz native pipeline
scripts/	
2bRADnative_processdata.R	Code to transpose 2bRAD data into Phylip format and calculate shared loci for reproducibility analysis
2bRAD_depth_stats.R	Code to calculate read depth statistics for 2bRAD data
2bRAD_clustthreshold_analysis.R	Code to generate results from clustering threshold tests with 2bRAD data
retabvcf.pl	Script to process 2bRAD data[2]
walkthrough/	
Epi_Rana_2bRAD_2019.txt	Detailed walkthrough of 2bRAD assembly[2]
Matz_ddRAD/	
scripts/	
ddRAD_matz_process_data.R	Code to transpose data into Phylip format

[1] Found here: https://docs.google.com/document/d/14LzChQSHRMghGaWL55EYsZ09Ve-3ncHIJzv6TLYvBcg/edit?hl=en_US
[2] Adapted from scripts found here: https://github.com/z0on/2bRAD_denovo

walkthrough/	
Matz_ddRAD_walkthrough.txt	Detailed walkthrough of ddRAD assembly[2]
**3_Data_analysis**	Directory containing analyses files
scripts/	
calculate_depth_stats.R	Code to calculate read depth statistics reported per individual
clust_threshold_processing.R	Code to combine clustering threshold results from 2bRAD and ddRAD into a single data file for **Fig. 3**
Concatenate_data_tree_to_nexus.txt	Bash code to create Nexus files with trees and sequence data for PAUP* input
unambiguous_changes_analyses	Files relevant for unambiguous change / binary recoded analyses
1-Permutation-Chisq-Analyses	Code to run permutation (chi-square test) analysis on binary-recoded data. Contains scripts/and Results/ directories and an R project file (.Rproj). R project is used to generate **Fig. S2**
2-Parse-ChgList-Analyses-Write-Tables<br>3-Parse-ApoList-Analyses<br>4-Parse-ApoList-Char-Quality-Chisq	Code to parse three types output PAUP* .log files: character change lists (ChgList), apomorphy lists (ApoList), and Chi-square analyses (Char-Quality-Chisq). All directories contain a scripts/ folder and an R project file (.Rproj)
**4_Data_visualization**	Directory containing files for producing all figures
data_files_input_into_scripts/	
2bRAD_shared_loci_replicates.csv	Data file with shared loci between replicate samples for 2bRAD (**Fig. 11**)
ddRAD_shared_loci_replicates.csv	Data file with shared loci between replicate samples for ddRAD (**Fig. 11**)
master.nexus	Master *Epipedobates* and *Rana* trees for plotting (**Figs. 6, 7, 10, & S1**)
Node_numbering_master_trees.png	Image for node standardization between R master tree and PAUP* output
Plot-Data-for-MS-FigS1.txt	Data file with unambiguous changes for binary-recoded datasets (**Figs. 7 & S1**)
readdepth_missingdata_snps.txt	Data file with average read depth per sample (calculated using vcfs; **Fig. 4**), and amounts and proportions of missing data (**Fig. 8**)
recoded_signonsig.txt	Data file with binary-recoded significant unambiguous changes (**Fig. 10**)
Retention_PIs.csv	Data file with retention indices and parsimony-informative sites (**Fig. 6**)
unambig_sums.txt	Data file with unambiguous changes (**Figs. 7 & S1)**
clust_threshold_data.txt	Data file with clustering threshold results (**Fig. 3**).

scripts/	
Fig3_Data_characterization.R	Code to construct **Fig. 3:** comparisons among clustering threshold values for ddRAD (iPyrad pipeline) and 2bRAD (Matz pipeline) data
Fig4_read_depth.R	Code to construct **Fig. 4**: average read depth per individual and read depth vs missing data
Fig6_Retention_index.R	Code to construct **Fig. 6:** parsimony-informative sites and retention indices
Fig7&S1_PAUP_analysis.R	Code to construct **Figs. 7 & S1:** proportions and numbers of unambiguous changes along branches of trees
Fig8_Missing_data.R	Code to construct **Fig. 8:** proportions of missing data
Fig9_Dollo_analysis.R	Code to construct **Fig. 9:** proportions of state changes from recoded datasets
Fig10_Recoded_significance_analysis.R	Code to construct **Fig. 10:** proportions of significant/non-sig changes
Fig11_a_Split_loci_files.ipynb	Code to split .loci files outputted by iPyrad for ddRAD data
Fig11_b_Shared_loci_replicates.ipynb	Code to calculate shared loci between replicate samples from ddRAD
Fig11_c_Shared_loci_replicates.R	Code to construct **Fig. 11:** shared loci between replicate samples

Chambersetal_SuppMats_Data

Directory	Description
**1_Bioinformatics**	
iPyrad_ddRAD/	All files relevant to ddRAD bioinformatics pipeline in iPyrad
clust_threshold/	Input and output files used to test clustering threshold values 80-95%
*_parameterfiles/	iPyrad parameter files
*_outfiles/	iPyrad output files
clust_threshold_summary_data/	Output data files from concatenating iPyrad stats files
sampling_depth/	Input and output files for different sampling depths (t1, t2, t3, total)
*_parameterfiles/	iPyrad parameter files
*_outfiles/	iPyrad output files
*.phy	Complete (full loci) datasets
*.snps.phy	SNP datasets
*_stats.txt	Output stats files from iPyrad
sampling_depth_summary_data/	Output data files from concatenating iPyrad stats files
iPyrad_2bRAD/	All files relevant to 2bRAD bioinformatics pipeline in iPyrad
sampling_depth/	Input and output files for different sampling depths (*t1*, *t2*, *t3*, *total*)
*_parameterfiles/	iPyrad parameter files
*_outfiles/	iPyrad output files
*.phy	Complete (full loci) datasets
*.snps.phy	SNP datasets
*_stats.txt	Output stats files from iPyrad
sampling_depth_summary_data/	Output data files from concatenating iPyrad stats files
Matz_2bRAD/	2bRAD bioinformatics pipeline files using Matz native pipeline
epi2brad_Matz/	Output files from *Epipedobates* 2bRAD bioinformatics assembly
clust_threshold/	Input and output files used to test clustering threshold values 80–95%
*_allsites	Complete (full loci) datasets
*_varsites	SNP datasets
rana2brad_Matz/	Output files from *Rana* 2bRAD bioinformatics assembly
clust_threshold/	Input and output files used to test clustering threshold values 80–95%
*_allsites	Complete (full loci) datasets
*_varsites	SNP datasets

summary_data/	
2brad_depth.txt	Concatenated read depth data
Matz_ddRAD/	
epiddrad_Matz/	Output files from *Epipedobates* ddRAD bioinformatics assembly
*_allsites	Complete (full loci) datasets
*_varsites	SNP datasets
ranaddrad_Matz/	Output files from *Rana* ddRAD bioinformatics assembly
*_allsites	Complete (full loci) datasets
*_varsites	SNP datasets
**2_Tree_reconstruction**	All files relevant to running tree reconstruction in RAxML
RAxML_input_phylip/	Directory containing Nexus files for RAxML tree reconstruction
2brad /	
ddrad/	
RAxML_output/	Directory containing output files from RAxML tree reconstruction
2brad/	Phylogenetic trees produced from 2bRAD data
ddrad/	Phylogenetic trees produced from ddRAD data
**3_Data_analysis**	Directory containing analyses files
missing_data_output/	Output from PAUP* missing data analysis; replicates removed afterwards for final calculations
sharedsites_retindex_PIs/	Files relevant for retention indices and parsimony-informative sites
input_files/	Nexus files for input into PAUP*; replicates removed (n=10)
output_files/	Output .log files from PAUP*
unambiguous_changes_analyses	Files relevant for unambiguous change / binary recoded analyses
1-Permutation-Chisq-Analyses	Code to run permutation (chi-square test) analysis on binary-recoded data. Contains scripts/and Results/ directories and an R project file (.Rproj). R project is used to generate **Fig. S2**
2-Parse-ChgList-Analyses-Write-Tables<br>3-Parse-ApoList-Analyses<br>4-Parse-ApoList-Char-Quality-Chisq	Code to parse three types output PAUP* .log files: character change lists (ChgList), apomorphy lists (ApoList), and Chi-square analyses (Char-Quality-Chisq). All directories contain a scripts/ folder and an R project file (.Rproj)
Nexus-Log-Files/	Input files for unambig. change analyses

Nexus-files-snps-original-10taxa	SNP dataset Nexus files containing PAUP* analysis block
Nexus-files-binary	Binary-recoded Nexus files containing PAUP* analysis block
Permutation-nexus-files-dollo	Binary-recoded Nexus files containing PAUP* analysis block for permutation tests
output_files/	Output files from unambiguous change / binary recoded analyses
Log-files-snps-original-FINAL/<br>Log-files-binary-dollo-FINAL/<br>Log-files-binary-unord-FINAL/	These directories contain three folders with output .log files for each PAUP* analysis performed: *-apo/ contains apomorphy lists; *-chg/ contains character change lists; *-diag/ contains character diagnostics. Analyses were performed on original SNP datasets, on binary-recoded datasets under Dollo parsimony and unordered
Permutation-Log-Files-dollo-diagnostics/	Output .log files from permutation tests, organized by patterns of present/absent sites
